# Switching transcription with bacterial RNA polymerase through photocaging, photorelease and phosphorylation reactions in the major groove of DNA†
†Electronic supplementary information (ESI) available: Contains detailed experimental procedures, characterization data and additional figures and gels. See DOI: 10.1039/c9sc00205g


**DOI:** 10.1039/c9sc00205g

**Published:** 2019-03-04

**Authors:** Zuzana Vaníková, Martina Janoušková, Milada Kambová, Libor Krásný, Michal Hocek

**Affiliations:** a Institute of Organic Chemistry and Biochemistry , Czech Academy of Sciences , Flemingovo nam. 2 , 16610 Prague 6 , Czech Republic . Email: hocek@uochb.cas.cz; b Department of Organic Chemistry , Faculty of Science , Charles University in Prague , Hlavova 8 , CZ-12843 Prague 2 , Czech Republic; c Dept. of Molecular Genetics of Bacteria , Institute of Microbiology , Czech Academy of Sciences , CZ-14220 Prague 4 , Czech Republic . Email: krasny@biomed.cas.cz

## Abstract

We report proof of principle biomimetic switching of transcription *in vitro* through non-natural chemical reactions in the major groove of DNA templates. Photocaged DNA templates containing nitrobenzyl-protected 5-hydroxymethyluracil or – cytosine permitted no transcription with *E. coli* RNA polymerase (OFF state). Their irradiation with 400 nm light resulted in DNA templates containing hydroxymethylpyrimidines, which switched transcription ON with a higher yield (250–350%) compared to non-modified DNA. Phosphorylation of templates containing 5-hydroxymethyluracil (but not 5-hydroxymethylcytosine) then turned transcription OFF again. It is the first step towards artificial bioorthogonal chemical epigenetics.

## Abstract 2

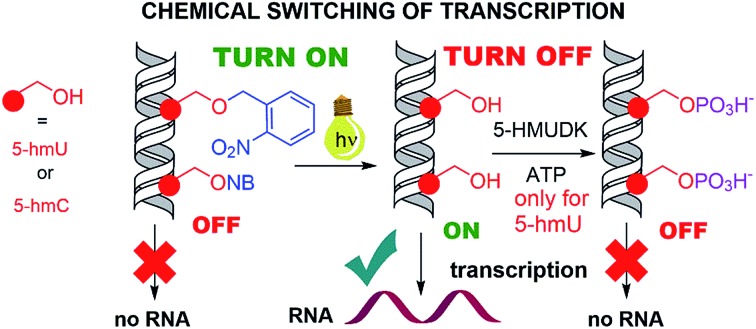
Biomimetic switching of *in vitro* transcription was developed by photochemical deprotection of photocaged 5hmU or 5hmC in template DNA (ON) and by enzymatic phosphorylation (OFF).

## 

Epigenetic modifications of DNA by 5-methylcytosine and its oxidized congeners, *i.e.* 5-hydroxymethyl- or 5-formylcytosine, regulate gene expression^1^–^5^ through enhancing or inhibition of binding of transcription factors (TFs) and RNA polymerases (RNAP) to genomic DNA^6^–^8^ or through modulation of chromatin properties.^9^,^10^ Natural DNA methylation and demethylation occurs during the differentiation of cells to switch on and off certain genes.^1^–^5^,^11^–^15^ Despite great progress in recent years, the biological roles of the different epigenetic modifications are not yet fully understood.^1^–^17^ On the other hand, there is a challenging opportunity to introduce some non-canonical modifications to DNA to explore their possible use in regulation of gene expression.^18^–^23^ We have reported a study of transcription of DNA templates bearing different non-natural modifications in the major groove by bacterial RNAPs and found that bulkier modifications inhibited transcription whereas some small modifications were tolerated and the modified DNA templates were still transcribed into RNA.^24^ We also found that DNA templates containing 5-hydroxymethyluracil (**U^hm^**), a rare natural base whose biological role is yet unknown,^25^–^28^ can enhance (up to 3.5 times) transcription depending on the promoter.^29^ We envisaged that some bioorthogonal chemical reactions in the major groove of DNA could be used to manipulate the bulkiness of the modification and we recently published the first paper on turning OFF transcription through a click reaction of 5-ethynyluracil in the major groove.^30^ Understanding of how nucleic acids can be modified and subsequently interact with RNAP is still in its infancy. Here we report a proof of principle one-way switch ON and OFF through photocaging, photochemical deprotection, and phosphorylation of 5-hydroxymethyluracil or – cytosine (**C^hm^**).

Photocaging of nucleic acids is frequently used for transient blocking of hybridization or other interactions which can be restored by photochemical release.^31^–^35^ We had recently reported the use of nitrobenzyl-37 or nitropiperonyl-caged^38^ 5-hydroxymethyluracil or 5-hydroxymethylcytosine^39^ for transient protection of DNA against the cleavage by restriction endonucleases whereas more bulky nitrophenylethyl-caged nucleotides were previously used as reversible chain terminators.^40^,^41^ Therefore, the nitrobenzyl photocaging and release of **U^hm^** and **C^hm^** was our first choice to set up a system that would allow to artificially switch transcription ON. In the opposite direction, we envisaged that phosphorylation of 5-hydroxymethyluracil by the natural 5-HMU DNA kinase (5-HMUDK)^42^–^44^ might be used to switch transcription OFF due to the increased bulkiness and negative charge of the phosphorylated **U^hm^**.

## Results and discussion

**Scheme 1:**
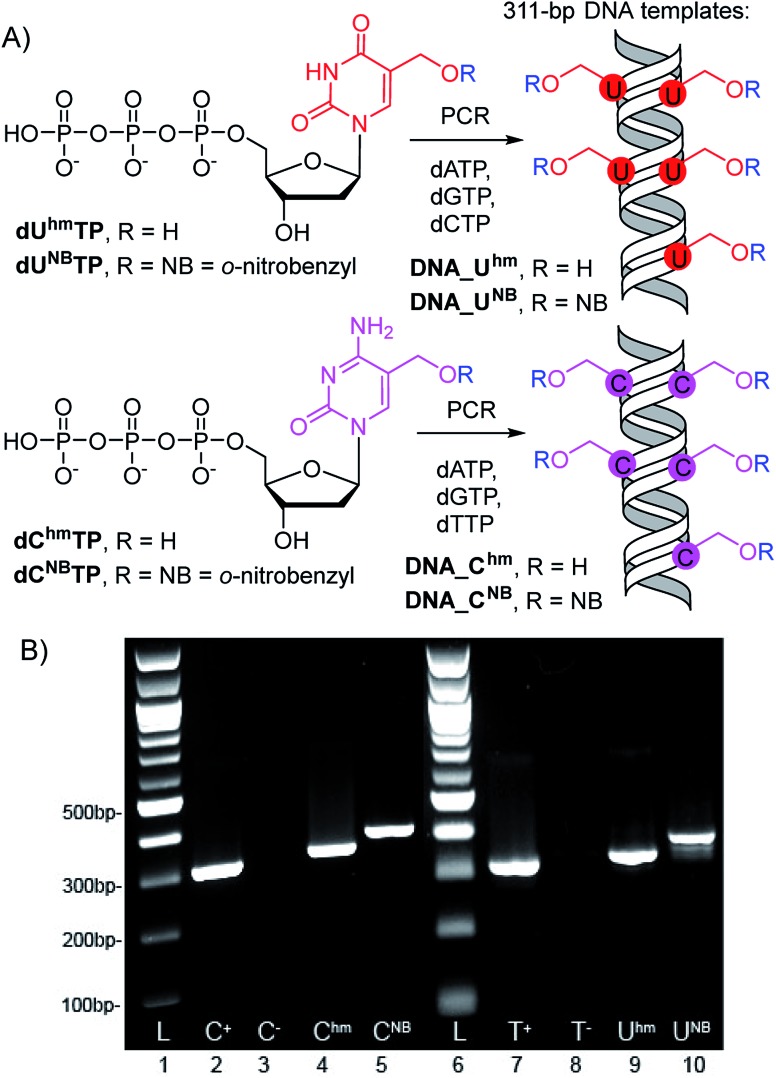
(A) PCR synthesis of the modified DNA templates, (B) agarose gel electrophoresis of the PCR products.

The 311-bp templates for transcription containing the Pveg promoter for transcription with *E. coli* RNAP were designed similarly as previously reported^30^,^45^ and were prepared by PCR using modified **dU^hm^TP**, **dU^NB^TP**,^37^**dC^hm^TP** or **dC^NB^TP**^39^ instead of the corresponding natural pyrimidine nucleotide (Scheme 1). In all cases, full length amplicons were obtained efficiently and after isolation were used as templates for *in vitro* transcription experiments.

**Fig. 1:**
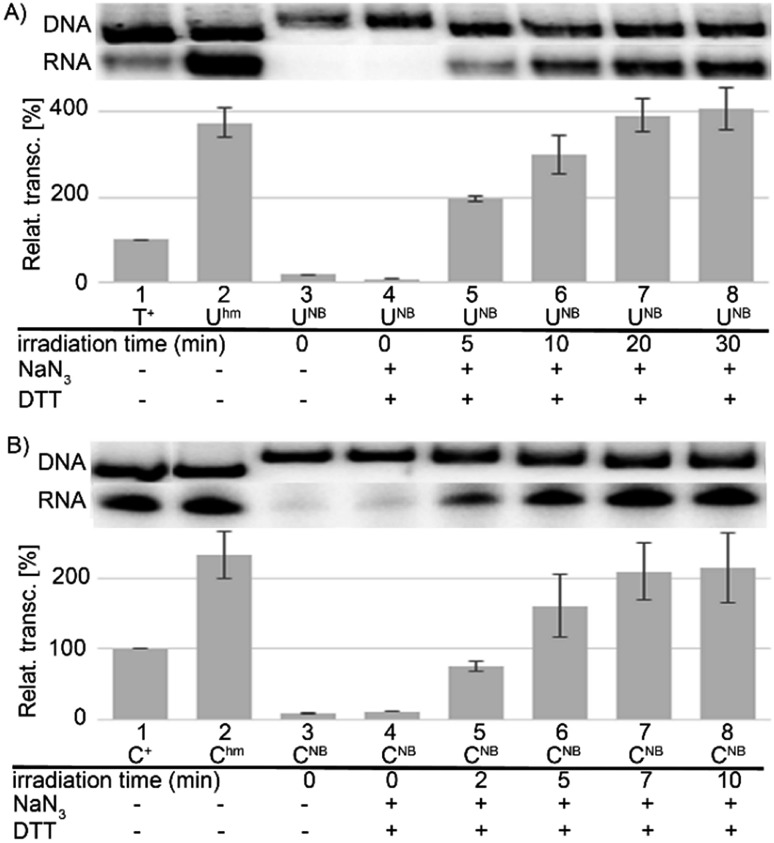
Kinetics of photochemical deprotection of NB-caged DNA templates [**DNA_U^NB^** (A), **DNA_C^NB^** (B)] monitored by transcription (lanes 3–8). Lanes 1 and 2 show control transcriptions from natural DNA and **DNA_U^hm^** or **DNA_C^hm^**, respectively. Representative primary data (DNA templates and RNA transcripts) are shown. The graphs in this and following Figures are averages of at 2–3 independent experiments ±SD. The time of irradiation and usage of two additives (DTT and NaN_3_) are indicated below the graphs.

In accord with previous work,^29^ DNA containing **U^hm^** or **C^hm^** displayed increased transcription compared to natural templates (*ca.* 350 or 220–250%, respectively), whereas templates containing the bulky photocaged bases **U^NB^** or **C^NB^** gave negligible transcription (<15%). We used ^32^P-labelled PCR products to accurately quantify the amounts of DNA templates because UV absorption or GelRed staining are not reliable for quantification of base-modified DNA.^29^ The photochemical deprotection of the photocaged templates (**DNA_U^NB^** or **DNA_C^NB^**) was performed using a 3 W photodiode with a maximum *λ* at 400 nm (in analogy to previous works^38^,^39^). In order to avoid DNA damage and absorption of light by nitrosobenzaldehyde, which is released as the byproduct,^46^ we used 1,4-dithiothreitol (DTT) and sodium azide as additives^47^ (see Fig. S6 in ESI† for the study of the influence of additives). At first, we carried out a simple kinetic study of irradiation of **DNA_U^NB^** or **DNA_C^NB^** for different reaction times, checked the completion of the deprotection of **DNA_U^NB^** by cleavage with REs^38^,^39^ (see Fig. S9 in ESI†) and then used them as templates for transcription. In control experiments, we irradiated the non-modified, as well as hydroxymethylated **DNA_U^hm^** or **DNA_C^hm^** templates to confirm that the irradiation had no effect on the non-photocaged DNA templates and their transcription. The kinetic study (Fig. 1, S10 and S12 in ESI†) showed that the irradiation of **DNA_U^NB^** (for 20 min) or **DNA_C^NB^** (for 10 min) released DNA templates and resulted in approximately the same level of transcription as the corresponding **DNA_U^hm^** or **DNA_C^hm^** templates (*ca.* 350 or 230%, respectively) indicating that the deprotection had been completed. This is in accord with our previous studies of the kinetics of photorelease using cleavage with restriction endonucleases as indicator of the photodeprotection.^38^,^39^ On the other hand, longer irradiation (>30 min, Fig. S10 and S12 in ESI†) led to a gradual decrease in transcription probably due to DNA damage.

**Scheme 2:**
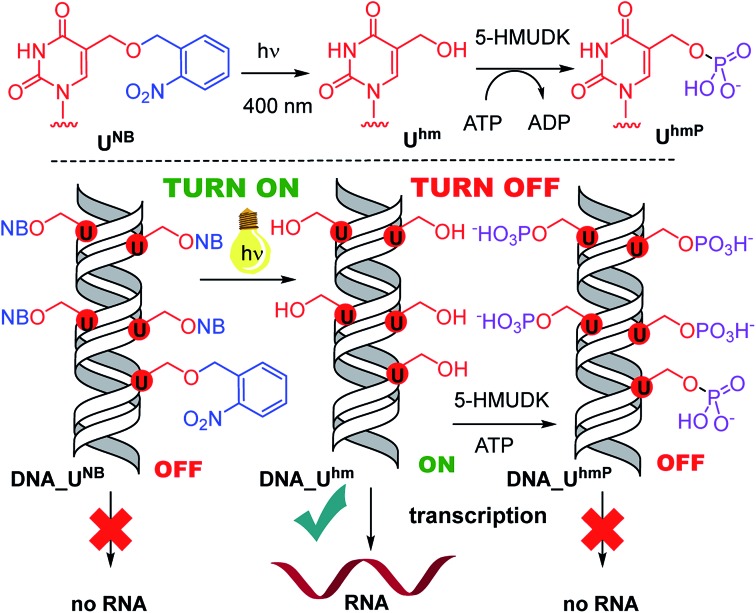
Switching transcription with photocaged DNA templates containing **U^hm^**.

**Fig. 2:**
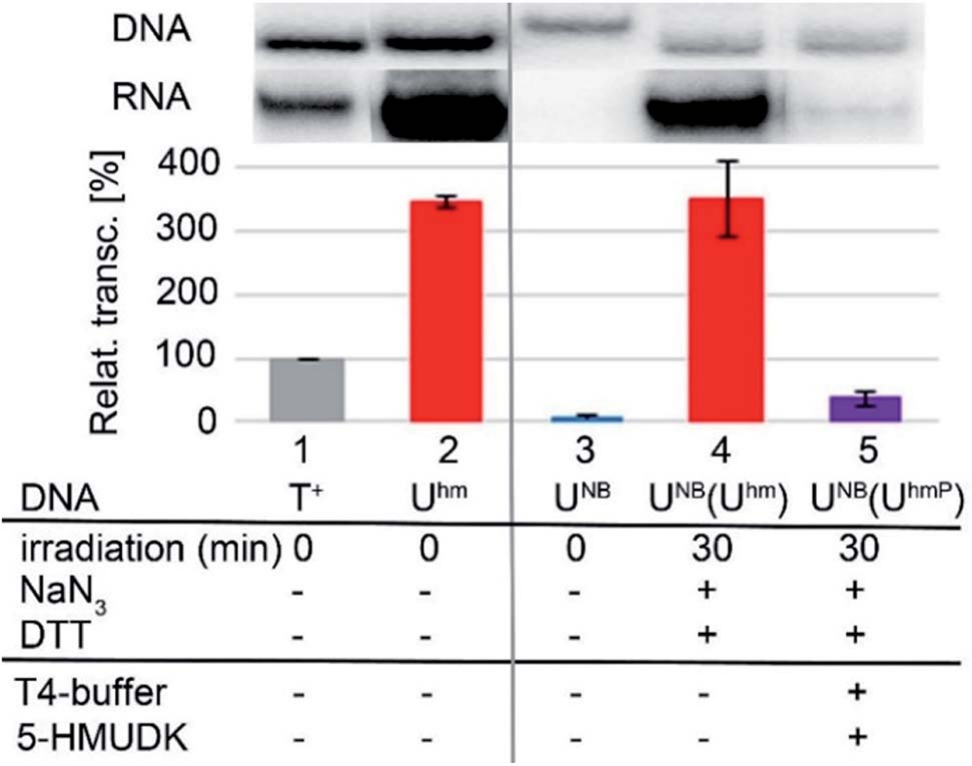
*In vitro* transcription from natural DNA (lane 1), **DNA_U^hm^** (2) and **DNA_U^NB^** (3) templates. Lane 4 shows transcription from **DNA_U^NB^** template after 30 min irradiation with *λ* = 400 nm. Lane 5 shows transcription from **DNA_U^NB^** template after irradiation followed by treatment with 5-HMUDK and ATP.

Next, we used the optimized conditions for a preparative experiment to turn transcription ON and OFF. Thus, the **DNA_U^NB^** template (which gives negligible transcription) was irradiated at 400 nm for 30 min and the resulting **DNA_U^hm^** gave rise to the expected 350% transcription increase. Then, phosphorylation of the deprotected **DNA_U^hm^** was performed in the presence of 5-HMUDK and ATP. Unlike in the photodeprotection of **U^NB^** to **U^hm^**,^38^,^39^ we could not use cleavage by REs as accurate measure of the yield of phosphorylation (see Scheme S3 and Fig. S13 in ESI†). Therefore, we proceeded directly to the transcription study and found that the resulting phosphorylated template **DNA_U^hmP^** supported only a low level of transcription (37% compared to the non-modified DNA template, 10% compared to the starting **DNA_U^hm^**), which indicates a significant (though not complete) switching OFF (Scheme 2, Fig. 2, see also Fig. S15A in ESI† for complete uncut gels).

**Scheme 3:**
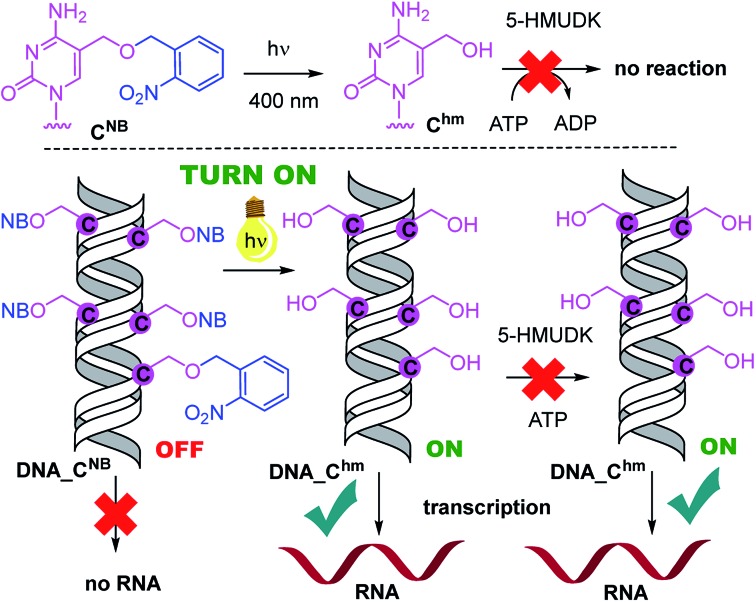
Switching transcription with photocaged DNA templates containing **C^hm^**.

**Fig. 3:**
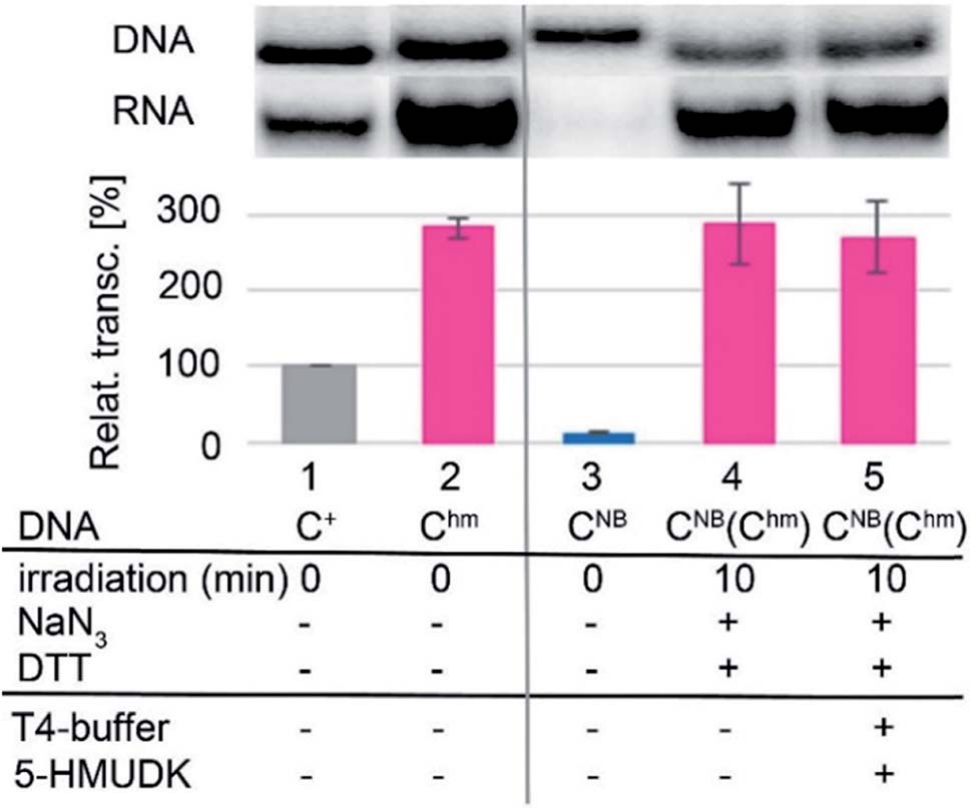
*In vitro* transcription from natural DNA (lane 1), **DNA_C^hm^** (2) and **DNA_C^NB^** (3) templates. Lane 4 shows transcription from **DNA_C^NB^** template after 10 min irradiation with *λ* = 400 nm. Lane 5 shows transcription from **DNA_C^NB^** template after irradiation followed by treatment with 5-HMUDK and ATP.

Analogously, the **DNA_C^NB^** template (which by itself gives negligible transcription) was irradiated at 400 nm for 10 min to yield the deprotected **DNA_C^hm^** template which restored its *ca.* 250% transcription level compared to natural DNA. However, since the 5-HMUDK specifically phosphorylates only **U^hm^**, the treatment of **DNA_C^hm^** with 5-HMUDK and ATP did not lead to a phosphorylated template and the transcription still proceeded at the same high level (Scheme 3, Fig. 3, see also Fig. S15B in ESI† for complete uncut gels).

In conclusion, we have demonstrated for the first time that bioorthogonal chemical reactions in the major groove of DNA can turn ON or OFF transcription with bacterial RNAP *in vitro*, similarly to the naturally-occurring DNA methylation and demethylation involved in epigenetic regulations of gene expression.^1^–^15^ Previously, we showed that DNA templates containing rare natural **U^hm^** or **C^hm^** supported transcription more efficiently than natural DNA, probably by facilitating the recruitment of RNAP to the promoter.^29^ Now we used nitrobenzyl-photocaging of the hydroxymethylated templates prevent transcription (OFF state), which can be then switched ON through photochemical deprotection using the relatively harmless 400 nm light (at least in low doses).^48^ In the case of **U^hm^**, the transcription can be switched OFF again by enzymatic phosphorylation. The decreased transcription from **DNA_U^hmP^** may indicate that the 5-HMUDK^42^–^44^ enzyme can serve as an epigenetic writer to inactivate genes which were accidentally activated due to oxidative formation **U^hm^** or as a defense against bacteriophages that contain DNA bearing this modification,^43^,^44^ however a further more detailed study will be needed to confirm this hypothesis. For photocaged **C^hm^**, the switch ON through photodeprotection proceeds in the same way as for photocaged **U^hm^**, however, the second switch OFF with 5-HMUDK does not work. Therefore, photocaged **U^hm^** in DNA templates function as a logic gate^49^–^51^ with binary transcriptional outputs of 0-1-0, whereas for **C^hm^** the outputs are 0-1-1. In principle, further switching could be envisaged by dephosphorylation of **DNA_U^hmP^** with a phosphatase or though enzymatic glycosylation of **DNA_C^hm^**.^52^ We are currently working on both of these reactions and, despite some initial unsuccessful experiments, we hope to be able to develop one or both of them to further extend the portfolio of transformations useful for regulation of transcription from modified DNA.

The presented new strategy of photocaging and release control of transcription in the major groove of DNA is conceptually different from previously known photocaging approaches^33^–^36^ where the photocaging groups interfere with Watson–Crick pairing of DNA bases preventing duplex formation and therefore the photocaged oligonucleotides (ONs) can only be prepared by chemical synthesis on solid support.^33^–^36^ In our approach, the photocaged oligonucleotides (ONs) form stable duplexes and can even be prepared enzymatically by direct polymerase incorporation of the modified nucleotides. We emphasize that the switching has so far only been demonstrated *in vitro* and any application *in cellulo* or even *in vivo* will be still very challenging (although both reactions are in principle biocompatible and bioorthogonal and we have recently reported transport of modified dNTPs and *in cellulo* incorporation of modified nucleotides into the genomic DNA^53^). However, this is the proof of principle, the first and essential step towards exciting artificial chemical regulations of gene expression. Follow up studies along these lines using these or other reactions^54^,^55^ are under way in our groups.

## Experimental

### Preparation of fully modified DNA and deprotection of **DNA_N^NB^** by light irradiation

Nitrobenzyl- and hydroxymethyl- modified DNA templates (**DNA_U^NB^**; **DNA_C^NB^**, **DNA_U^hm^**; **DNA_C^hm^**) containing specific Pveg promoter region were synthesized in the presence of either *NON*-labelled or ^32^*P-labelled* primers (*Prim^FOR^ –*^32^*P* and *Prim^REV^ –*^32^*P*) by PCR reaction under the conditions reported in ESI (ESI Section 2.3.1–2.3.3†). For the study of deprotection of photolabile nitrobenzyl protecting groups, the purified NB-modified DNAs (**DNA_U^NB^**; **DNA_C^NB^**) were diluted to the final concentration of approx. 20 ng μL^–1^ and used as a stock for irradiation experiments. Approx. 240 ng of stock nitrobenzyl-modified DNA (**DNA_U^NB^** or **DNA_C^NB^**) was irradiated by light from different photodiodes (355 nm, 365 nm or 400 nm) in the particular time intervals (ESI Section 4.2.†). The samples were irradiated either without additives or in the presence of NaN_3_ and DTT. The irradiated DNA, as well as natural or hm-modified or NB-modified DNA right after PCR, were used as templates for an *in vitro* transcription assay (ESI Section 4.†).

### General procedure for transcription studies of prepared DNA

Transcription studies of prepared DNA templates were performed with RNA polymerase (RNAP) from *Escherichia coli* (EcoRNAP) – a holoenzyme complexed RNAP with σ^70^. The resulted transcripts (RNA) were about 145 nucleobases long. Multiple round *in vitro* transcription assays were performed essentially as described.^29^,^30^ The experiments were carried out in total volume 10 μL with 5 ng of DNA template. The reactions proceeded for 10 min at 37 °C after previous preheating of reaction mixture without NTPs. For visualization of prepared RNA product, the transcription was performed in the presence of [α-^32^P] UTP. The reactions were stopped by the addition of 10 μL of formamide stop solution. The products of transcription were checked by running of 7% polyacrylamide gels. After scanning of exposed gels, the scanned gels were analysed with Quantity One program (BIORAD). For a quantification of relative transcriptions, the transcript signals were normalized to DNA template signals. Signals of transcriptions of modified DNA templates were normalized to the signal of natural DNA (T^+^ or C^+^), which was set as 100%. Two–three independent experiments were performed (ESI Section 4.†).

### Phosphorylation of hm-modified DNA with 5-HMUDK

Conditions for phosphorylation of hydroxymethyl-moieties on DNA were optimized on **DNA_U^hm^** synthesized in the presence of **dU^hm^TP** by PCR. Hydroxymethyl-modified DNA was incubated with different amount of 5-HMUDK (20U; 1.2 μL or 18U; 0.9 μL or 12U; 0.6 μL) at 37 °C for 30 min. The purified phosphorylated DNA (**DNA_U^hmP^**) along with natural DNA, which was exposed to the same conditions of phosphorylation were used as templates for transcription studies (ESI Section 5.†).

### DNA sample preparation for a study of switching ON and OFF transcription

For a study of switching ON and OFF transcription, the modified DNA templates were synthesized in the presence of ^32^*P-labelled* primers by PCR reaction under the reported conditions (ESI Section 2.3.1–2.3.3†). Purified **DNA_U^NB^** (cca 240 ng) was irradiated in the presence of additives [1 μL of (1 mM) NaN_3_ and 1 μL of (50 mM) DTT] with UV lamp (3 W, 400 nm) during 30 min in 1.5 mL Eppendorf tube at room temperature (Scheme S5A, Fig. S15A lane 10 in ESI†). The irradiation experiments were repeated in six portions. After irradiation, all six portions were mixed together and non-purified previously irradiated DNA (400 ng) was incubated under optimized conditions with 5-HMUDK (0.3 μL) in 1× T4 DNA Ligase Reaction Buffer at 37 °C for 30 min (Scheme S5A, Fig. S15A lane 12 in ESI†). As a control of phosphorylation, non-irradiated hydroxymethyl-modified DNA (synthesized by PCR in the presence of **dU^hm^TP**) incubated with 5-HMUDK under the same conditions was considered. As a control of selective phosphorylation, natural DNA, **DNA_C^hm^** and irradiated **DNA_C^NB^** (irradiated under the same conditions as **DNA_U^NB^** in time interval 10 min) were also incubated with 0.3 μL of 5-HMUDK at 37 °C during 30 min. In all cases, the DNA right after the reactions were used as templates for the multiple-round *in vitro* transcription assays (ESI Section 6.†).

## Conflicts of interest

There are no conflicts to declare.

## Supplementary Material

Supplementary information
