# Supplementary material for: Switching transcription with bacterial RNA polymerase through photocaging, photorelease and phosphorylation reactions in the major groove of DNA
Source: Chem Sci. 2019 Mar 4;10(14):3937–42. doi: 10.1039/c9sc00205g (PMC6457204; doi:10.1039/c9sc00205g)
Supplement: Supplementary file 1 [file SC-010-C9SC00205G-s001.pdf]

Electronic Supplementary Material (ESI) for Chemical Science.
This journal is © The Royal Society of Chemistry 2019

Electronic Supporting Information

For

Switching transcription with bacterial RNA polymerase through photocaging, photorelease and phosphorylation reactions in the major groove of DNA template

Zuzana Vaníková,[a,b] Martina Janoušková,[c] Milada Kambová,[c] Libor Krásný*[c] and Michal Hocek*[a,b]

[a] *Institute of Organic Chemistry and Biochemistry*
Academy of Sciences of the Czech Republic
Flemingovo nam. 2, 16610 Prague 6, Czech Republic
E-mail: hocek@uochb.cas.cz
Homepage: http://www.uochb.cas.cz/hocekgroup

[b] *Department of Organic Chemistry, Faculty of Science,*
Charles University in Prague,
Hlavova 8, CZ-12843 Prague 2, Czech Republic

[c] *Dept. of Molecular Genetics of Bacteria, Institute of Microbiology*
Academy of Sciences of the Czech Republic
CZ-14220 Prague 4, Czech Republic
E-mail: krasny@biomed.cas.cz
Homepage: http://www.biomed.cas.cz/mbu/krasny/

Experimental part

Structures of dNTPs, epigenetic nucleoside triphosphates (**dU^{hm}TP**, **dC^{hm}TP**) and nitrobenzyl modified nucleoside triphosphates (**dU^{NB}TP**, **dC^{NB}TP**), used for the synthesis of fully modified DNAs are shown in ***Figure S1***.

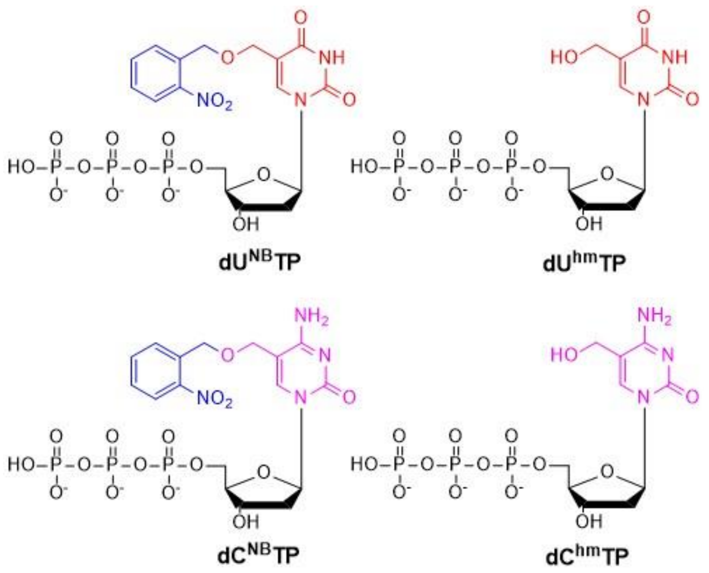

Figure S1: Structures of used triphosphates

1. General remarks

Synthetic oligonucleotides (*NON-labelled* primers) were purchased from GeneriBiotech. Natural nucleoside triphosphates (dATP, dCTP, dGTP, dTTP) were bought from New England Biolabs. **dU^{hm}TP**, **dC^{NB}TP** and **dU^{NB}TP** were prepared according to published procedure.[1,2] **dC^{hm}TP** was purchased from TriLink Biotechnologies. KOD XL DNA polymerase was purchased from Merck and Taq DNA polymerase for ThermoPol buffer were purchased from New England Biolabs. Restriction enzymes AluI and RsaI were purchased from New England Biolabs. All PCR products and final DNA templates were purified with Agencourt AMPure XP magnetic particles (Beckman Coulter Life Science - GE Healthcare).

2. Synthesis of fully HM- and NB-modified DNA templates

Table S1: *List of oligonucleotides used for synthesis of 311-mer*

**Oligonucleotide-**	**Sequence (5´→3´)**	**Length**
**99-mer ss oligos**		
Insert_99_EcoRI[a]	GAC**GAATTC**AGCCATATATCCTCTGGCTAATAGGACTACTTCTAAT CTGTACAGCAGATCCATACGCCTGGACAGGCAATCAGGCTAGAG **GAATTC**GTG	99-mer
Insert_99_EcoRI_REV[a]	CAC**GAATTC**CTCTAGCCTGATTGCCTGTCCAGGCGTATGGATCTG CTCGTACAGATTAGAAGTAGTCCTATTAGCCAGAGGATATATGGC T**GAATTC**GTC	99-mer
**PRIMERs**		
Prim ^{FOR}	TTCAGCCATATATCCTCTGGCTAATAGG	28-mer
Prim ^{REV}	GGAGAGCGTTCACCGACAAACAACAG	26-mer
**TEMPLATE**[b,c]		
Temp ^{Pveg2}	TTCAGCCATATATCCTCTGGCTAATAGGACTACTTCTAATC TGTACGAGCAGATCCATACGCCTGGACAGGCAATCAGGC TAGAGGAATTC*TATTTGACAAAAATGGGCTCGTGTTGTAC AATAAATGT*GTCTAAGCTTGGGTCCCACCTGACCCCATGCC GAACTCAGAAGTGAAACGCCGTAGCGCCGATGGTAGTGTG GGGTCTCCCCATGCGAGAGTAGGGAACTGCCAGGCATCA AATAAAACGAAAGGCTCAGTCGAAAGACTGGGCCTTTCGT TTTATCTGTTGTTTGTCGGTGAACGCTCTCC	311-mer

[a] bold sequence in oligo is site for cleavage with EcoRI

[b] primer sequences in template are underlined

[c] promoter sequence of template is underlined in italic

2.1. Preparation of plasmid with 311-mer region containing specific promotor region

The 1177 plasmid containing the P*veg* promoter[3] was used to clone a 99 bp DNA sequence upstream of the promoter. The sequence was inserted to facilitate subsequent PCR reaction with modified nucleotides. First, two complementary strands of comercially available 99-mer oligos **Insert_99_EcoRI** (100 uL of 0.1 mM) and **Insert_99_EcoRI_REV** (100 uL of 0.1 mM) were annealed under the following conditions: 95°C 5 min; gradient from 95°C to 25°C (change in

temperature every 2s). To check annealed DNA, 2% agarose gel stained by GelRed was run. (***Figure S1A***)

The prepared 99-mer dsDNA was then cleaved with EcoRI restriction endonuclease to create sticky ends and ligated into the 1177 plasmid into the EcoRI site at the upstream edge of the promoter. Such prepared plasmid was transformed into *E.coli DH5*α and colonies with inserts were identified and verified by sequencing, yielding the final construct LK2130.

2.2. Enzymatic synthesis of template (*Temp*^{Pveg2})

The 311-mer (***Temp***^{Pveg2}) template was prepared by PCR with forward (***Prim***^{FOR}) and reverse (***Prim***^{REV}) *NON-labelled* primers from plasmids containing specific promoter regions cloned in p770 between *Eco*RI and *Hind*III sites.[3,4] One PCR reaction mixture (20 µL) contained Taq DNA polymerase for ThermoPol buffer (New England Biolabs; 5000 U/mL; 1.2 µL) with ThermoPol buffer (2 µL), natural dNTPs (4 mM; 1.125 µL), primers (20 µM; 3 µL; ***Prim***^{FOR} and 20 µM; 3 µL; ***Prim***^{REV}) and appropriate plasmid template (42 ng). The reaction was performed in total volume 160 µL (8x20 µL). Forty PCR cycles were run in the thermal cycler under the following conditions: preheating for 3 minutes at 94°C, denaturation for 1 minute at 94°C, annealing for 1 minute at 68°C, extension for 1.5 minutes at 75°C, followed by final extension step of 5 minutes at 75°C. 160 µL of PCR reaction mixture was combined into one Eppendorf vial and PCR product was purified using Agencourt AMPure XP magnetic particles. In the last step of purification, the product was eluted with 200 µL of MilliQ water at the final concentration of 132 ng/µL of ***Temp***^{Pveg2}. After purification, 180 ng of DNA was loaded on a control 1.3% agarose gel stained with GelRed (Biotium) and analyzed in 0.5xTBE buffer. The sequence of template was confirmed by DNA sequencing. (***Figure S1B***)

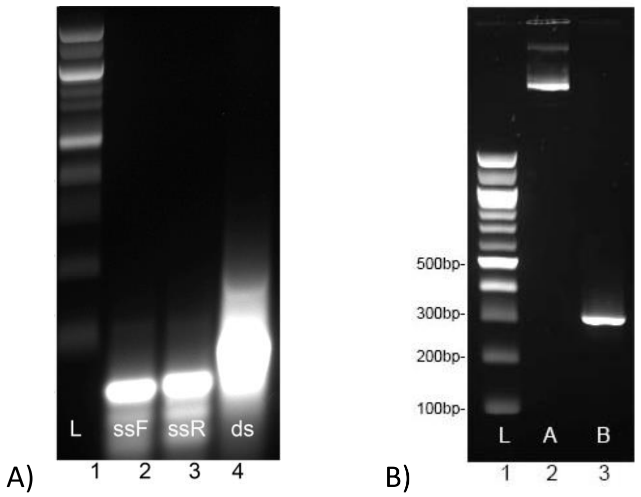

Figure S1: A) Agarose gel analysis of annealed inserts: Lane 1 (**L**): ladder (commercial mix of dsDNA with the specific length); lane 2 (**ssF**): oligo **Insert_99_EcoRI**; lane 3 (**ssR**): oligo **Insert_99_EcoRI_REV**; lane 4 (**ds**): annealed product B) Agarose gel analysis of PCR template product amplified by Taq DNA polymerase. Lane 1 (**L**): ladder (commercial mix of dsDNA with the specific length); lane 2 (**A**): plasmid, used as a template for amplification reaction; lane 3 (**B**): PCR product of template - $\boldsymbol{Temp^{Pveg2}}$

2.3. Synthesis of modified DNA templates

To prepare modified templates for transcription, PCR reactions were performed in a total final volume of 20 µL. Natural DNAs (positive controls on agarose gels), which were used as a controls for the DNA transcription experiments, were in all cases synthesized according to a procedure for $\mathbf{U^{hm}}$-modified or $\mathbf{C^{hm}}$-modified DNA without any additives and in the presence of all natural dNTPs (4 mM; 0.75 µL). (***Figures S2***)

All samples were prepared in the presence of either *NON-labelled* ($\boldsymbol{Prim^{FOR}}$ and $\boldsymbol{Prim^{REV}}$) or *^{32}P-labelled* ($\boldsymbol{Prim^{FOR} - {}^{32}P}$ and $\boldsymbol{Prim^{REV} - {}^{32}P}$) primers under the same conditions. The ^{32}P-labelling of primers was achieved by the transfer of the terminal phosphate from (γ)-^{32}P-dATP to the 5´-end of *NON-labelled* primers by T4 polynucleotide kinase.

Forty PCR cycles were run in the thermal cycler under the conditions described in protocol. PCR products of each type were then purified with Agencourt AMPure XP magnetic particles. The concentration of purified samples was measured by the NanoDrop spectrophotometr. All samples were diluted to a final concentration of 20 ng/uL according NanoDrop and used for transcription experiments. The exact quantity of DNA for transcription was analysed based on

the DNA signals (fluorescence and/or radioactivity) on a gel. Control 1.3% agarose gels were stained with GelRed (Biotium) in the case of *NON-labelled* samples and *^{32}P-labelled* DNA. (***Figure S2***) Moreover for the purpose of transcription, *^{32}P-labelled* DNA was determined throught 7% PAGE gels exposed to Fuji MS phosphor storage screens. (***Figure S9***; ***Figure S11***)

2.3.1. Synthesis of U^{hm}-modified DNA

The PCR reaction mixture (20 µL) contained KOD XL DNA polymerase (*Merck; Novagen;* 2.5 U/µL; 1.2 µL), natural dNTPs (dCTP, dGTP and dATP; 4 mM; 0.75 µL), **dU^{hm}TP** (4 mM; 1.5 µL), 311-mer template (40 ng/µL; 0.9 µL; ***Temp^{Pveg2}***), and primers - either *NON-labelled* or *^{32}P-labelled* (20 µM; 3 µL; ***Prim^{FOR}*** and 20 µM; 3 µL; ***Prim^{REV}***) in 10x reaction Buffer for KOD XL (2 µL) supplied by the manufacturer. Forty PCR cycles were run in the thermal cycler under the following conditions: preheating for 3 minutes at 94°C, denaturation for 1 minute at 94°C, annealing for 1 minute at 68°C, extension for 1.5 minutes at 75°C, followed by final extension step of 5 minutes at 75°C. ***(Figure S2A)***.

2.3.2. Synthesis of U^{NB}-modified DNA

The PCR reaction mixture (20 µL) contained KOD XL DNA polymerase (*Merck; Novagen;* 2.5 U/µL; 1.5 µL), natural dNTPs (dCTP, dGTP and dATP; 4 mM; 0.75 µL), **dU^{NB}TP** (4 mM; 2 µL), 311-mer template (40 ng/µL; 0.9 µL; ***Temp^{Pveg2}***) and primers - either *NON-labelled* or *^{32}P-labelled* (20 µM; 3 µL; ***Prim^{FOR}*** and 20 µM; 3 µL; ***Prim^{REV}***) in 10x reaction Buffer for KOD XL (2 µL) supplied by the manufacturer. Forty PCR cycles were run in the thermal cycler under the following conditions: preheating for 3 minutes at 94°C, denaturation for 1 minute at 94°C, annealing for 1 minute at 62°C, extension for 2 minutes at 75°C, followed by a final extension step of 7 minutes at 75°C. ***(Figure S2B)***.

2.3.3. Synthesis of C^{hm} and C^{NB}-modified DNA

The PCR reaction mixture (20 µL) contained KOD XL DNA polymerase (*Merck; Novagen;* 2.5 U/ µL; 1.2 µL), natural dNTPs (dTTP, dGTP and dATP; 4 mM; 0.75 µL), **dC^{hm}TP** (4 mM; 1.5 µL) or dC^{NB}TP (4 mM; 2 µL), 311-mer template (40 ng/µL; 0.9 µL; ***Temp^{Pveg2}***) and primers - either *NON-labelled* or *^{32}P-labelled* (20 µM; 3 µL; ***Prim^{FOR}*** and 20 µM; 3 µL; ***Prim^{REV}***) in 10x reaction Buffer for KOD XL (2 µL) supplied by the manufacturer. Forty PCR cycles were run in

the thermal cycler under the following conditions: preheating for 3 minutes at 94°C, denaturation for 1 minute at 94°C, annealing for 1 minute at 68°C, extension for 1.5 minutes at 75°C, followed by a final extension step of 5 minutes at 75°C. ***(Figure S2C)***

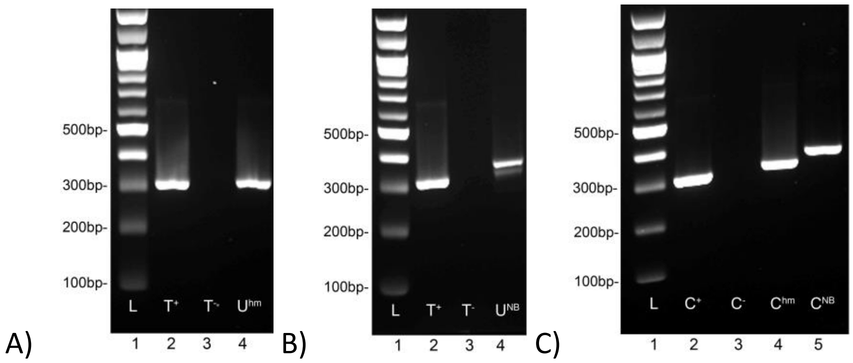

Figure S2: Gel analysis of PCR products that were either *NON-labelled* **A**), **B**) and **C**) or *^{32}P-labelled*. In gel **A**), the order of samples is as follows: lane 1 (**L**): ladder (mix of commercial dsDNA with specific length); lane 2 (**T^{+}**): PCR was run in the presence of natural dNTPs; lane 3 (**T^{-}**): PCR was run in the presence of dCTP, dGTP, dATP; lane 4, (**U^{hm}**): PCR was run in the presence of three natural dNTPs (dCTP, dGTP, dATP) and **dU^{hm}TP**; In gel **B**), the order of samples is as follows: lane 1 (**L**): ladder (mix of commercial dsDNA with specific length); lane 2 (**T^{+}**): PCR was run in the presence of natural dNTPs; lane 3 (**T^{-}**): PCR was run in the presence of dCTP, dGTP, dATP; lane 4, (**U^{NB}**): PCR was run in the presence of three natural dNTPs (dCTP, dGTP, dATP) and **dU^{NB}TP.** In gel **C**), the order of samples is as follows: lane 1 (**L**): ladder (mix of commercial dsDNA with specific length); lane 2 (**C^{+}**): PCR was run in the presence of natural dNTPs; lane 3 (**C^{-}**): PCR was run in the presence of dTTP, dGTP, dATP; lane 4, (**C^{hm}**): PCR was run in the presence of three natural dNTPs (dTTP, dGTP, dATP) and **dC^{hm}TP**; lane 5, (**C^{NB}**): PCR was run in the presence of three natural dNTPs (dTTP, dGTP, dATP) and **dC^{NB}TP**.

3. Study of cleavage of modified DNA with restriction endonucleases AluI and RsaI

A)

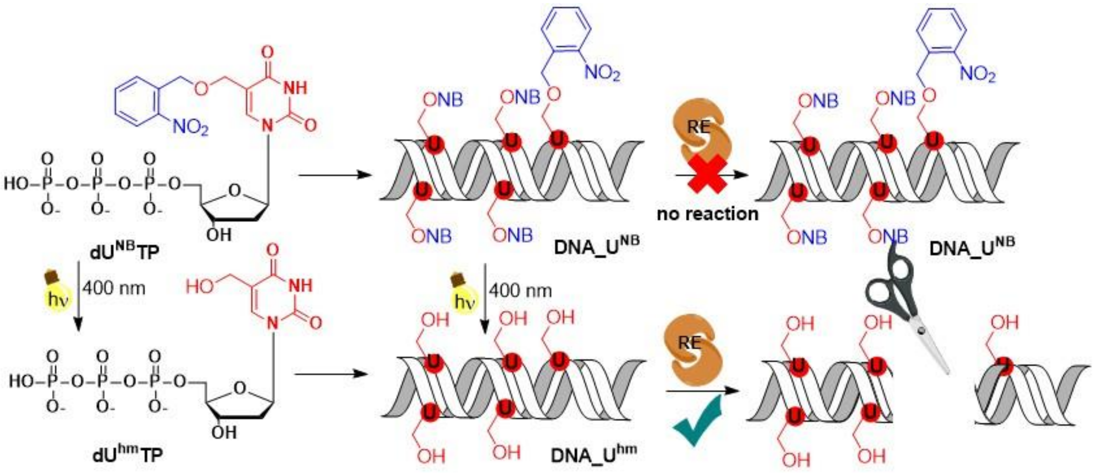

B)

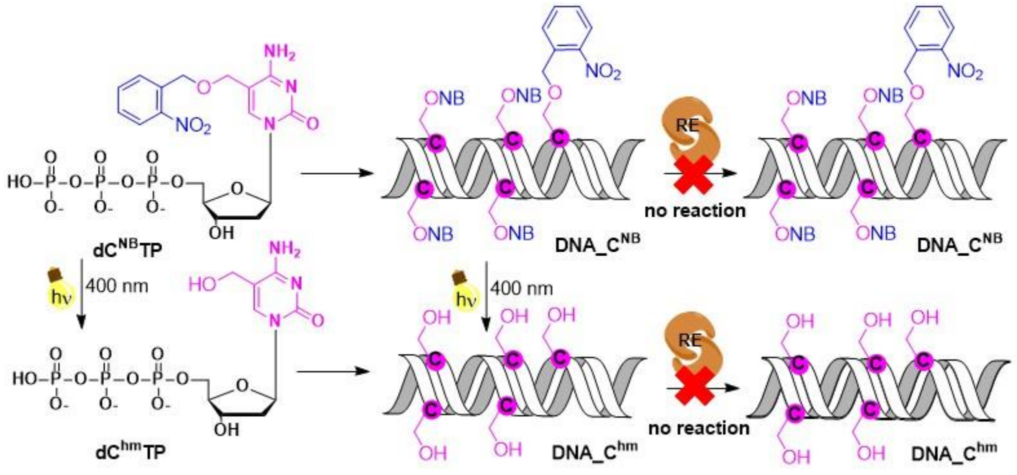

Scheme S1: Scheme of cleavage of modified DNA with REs

Hydroxymethyl (hm-) and nitrobenzyl- (NB-) modified DNA prepared according the reported protols (section *2.3.1* - *2.3.3*) were tested for their cleavage by restriction endonuclease, either AluI or RsaI.

3.1. Study of cleavage of modified DNA with AluI

Approx. 100 ng of either natural, hm-modified or NB-modified DNAs were incubated at 37 °C with addition of CutSmart Buffer (1 µL) and restriction enzyme AluI (1.8 µL) during 1 hour. The products of cleavage were then monitored with 1.3% agarose gel stained with GelRed. (***Scheme S1, Figure S3***)

3.2. Study of cleavage of modified DNA with RsaI

Approx. 100 ng of either natural, hm-modified or NB-modified DNAs were incubated at 37 °C with addition of CutSmart Buffer (1 µL) and restriction enzyme RsaI (1.4 µL) for 1 hour. In the case of samples, which were synthesized in the presence of **dC^{X}TP**s, 1.8 µL of RsaI enzyme was used for a cleavage DNA (***Figure S3***). The products of cleavage were then monitored with 1.3% agarose gel stained with GelRed. (***Figure S3***)

Despite the fact that the short 40-mer hm-modified DNA (synthesized in the presence of **dC^{hm}TP**) was easily cleaved with RsaI[2], hydroxymethyl- modification in long DNA coming from **dC^{hm}TP** was not tolerated with restriction enzymes - AluI or RsaI. Because of that, the kinetic study of deprotection was firstly performed just with modified DNA synthesized in the presence of modified **dU^{X}TP** (X=NB or hm).

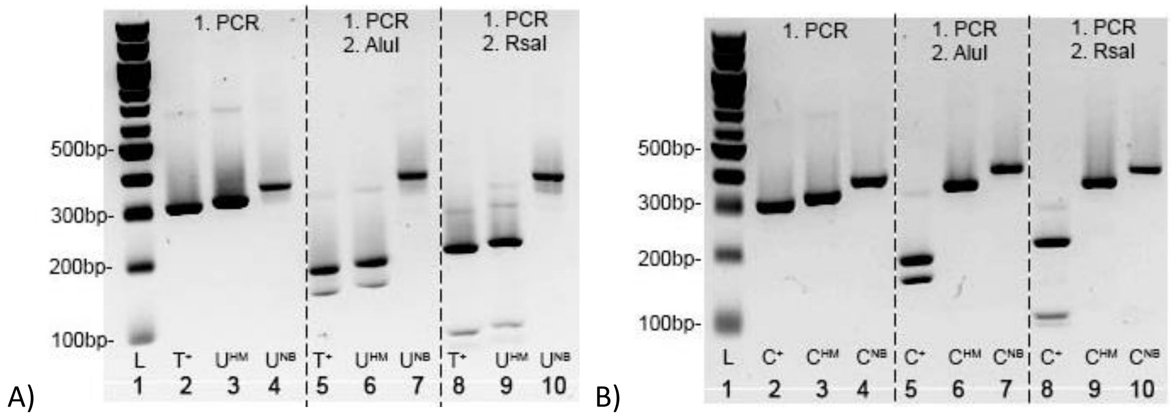

Figure S3: Study of cleavage of modified DNA with restriction endonucleases AluI and RsaI

In gel **A)** the order of samples is as follows: lane 1 (**L**): ladder (mix of commercial dsDNA with specific length); lane 2 (**T$^+$**): PCR product synthesized in the presence of natural dNTPs; lane 3 (**U^{HM}**): PCR product synthesized in the presence of three natural dNTPs (dCTP, dGTP, dATP) and **dU^{hm}TP**; lane 4 (**U^{NB}**): PCR product synthesized in the presence of three natural dNTPs (dCTP, dGTP, dATP) and **dU^{NB}TP**; lane 5 (**T$^+$**): natural DNA cleaved with restriction enzyme AluI; lane 6 (**U^{HM}**): hm-modified DNA cleaved with restriction enzyme AluI; lane 7 (**U^{NB}**): NB-modified DNA cleaved with restriction enzyme AluI; lane 8 (**T$^+$**): natural DNA cleaved with restriction enzyme RsaI; lane 9 (**U^{HM}**): hm-modified DNA cleaved with restriction enzyme RsaI; lane 10 (**U^{NB}**): NB-modified DNA cleaved with restriction enzyme RsaI. In gel **B)**: the order of samples is as follows: lane 1 (**L**): ladder (mix of commercial dsDNA with specific length); lane 2 (**C$^+$**): PCR product synthesized in the presence of natural dNTPs; lane 3 (**C^{HM}**): PCR product synthesized in the presence of three natural dNTPs (dTTP, dGTP, dATP) and **dC^{hm}TP**; lane 4 (**C^{NB}**): PCR product synthesized in the presence of three natural dNTPs (dTTP, dGTP, dATP) and **dC^{NB}TP**; lane 5 (**C$^+$**): natural DNA cleaved with restriction enzyme AluI; lane 6 (**C^{HM}**): hm-modified DNA cleaved with restriction enzyme AluI; lane 7 (**C^{NB}**): NB-modified DNA cleaved with restriction enzyme AluI; lane 8 (**C$^+$**): natural DNA cleaved with restriction enzyme RsaI; lane 9 (**C^{HM}**): hm-modified DNA cleaved with restriction enzyme RsaI; lane 10 (**C^{NB}**): NB-modified DNA cleaved with restriction enzyme RsaI.

4. Study of transcription for hm- and NB-modified DNA templates

Subsequently, either natural or hm-modified or NB-modified DNAs were tested in multiple round transcriptions (***Scheme S2***). Transcriptions were performed with RNA polymerase (RNAP) from *Escherichia coli* (EcoRNAP) - a holoenzyme complexed RNAP with σ^{70}, that is commercially available (New England BioLabs). Transcription from the promoter was ended at an intrinsic terminator and the transcripts (RNA) were about 145 bases long. The hydroxymethyl modified **DNA** displayed an enhancing effect on transcription as reported

before[4, 5]. On the other hand, in the case of nitrobenzyl modified DNA, the inhibitory effect of the bulky-NB modification on transcription was observed.

4.1. General remarks for experiments of transcription

4.1.1. Quantification of DNA

DNA templates were quantified as described[4, 5] unless stated otherwise. DNA concentration was measured with a NanoDrop spectrophotometr and subsequently diluted to 5 ng/µL in water. 50 ng of *NON*-labelled DNA was analyzed on a 1.5% agarose gel dyed with 10,000x diluted GelRed (Biotium). DNA was visualized by UV light. 20-50 ng of ^{32}P labeled DNA was analyzed on a 5% PAA gel without urea and subsequently dried and exposed to Fuji MS phosphor storage screens and scanned with a Molecular Imager FX (BIORAD). The results were analyzed with Quantity One program (BIORAD).

4.1.2. Multiple round transcriptions

Multiple round *in vitro* transcription assays were performed essentially as described[4] unless stated otherwise. The experiments were carried out in 10 µL with 5 ng template, 100 mM Tris pH 8, 125 mM $MgCl_2$, 50 mM DTT, 90 mM KCl, 30 nM RNA polymerase holoenzyme from *Escherichia coli* (EcoRNAP σ70) purchased from New England Biolabs and NTPs (200 µM ATP, 1000 µM GTP, 200 µM CTP, 10 µM UTP plus 3.7 kBq [α-32P] UTP). The samples were preheated for 10 min at 37°C and started with NTPs. The reactions were allowed to proceed for 10 min at 37°C. Subsequently, the reactions were stopped by the addition of 10 µl of formamide stop solution (95% formamide, 20 mM EDTA, pH 8.0). 10 µL of each sample was loaded onto 7% polyacrylamide gels. The gels were dried and exposed to Fuji MS phosphor storage screens and scanned with a Molecular Imager FX (BIORAD) and analysed with Quantity One program (BIORAD).

4.1.3. Quantification of relative transcriptions

Firstly, the transcript signals (background was subtracted) were normalized to DNA template signals. Subsequently, signals of transcriptions of non-modified and modified DNA templates were normalized to the signal of natural DNA (T^+ or C^+), which was set as 100 % (T^+ or C^+ -

transcription of nonmodified DNA diluted in water). At least three independent experiments were performed.

4.2. Kinetic study of deprotection for NB-modified DNA

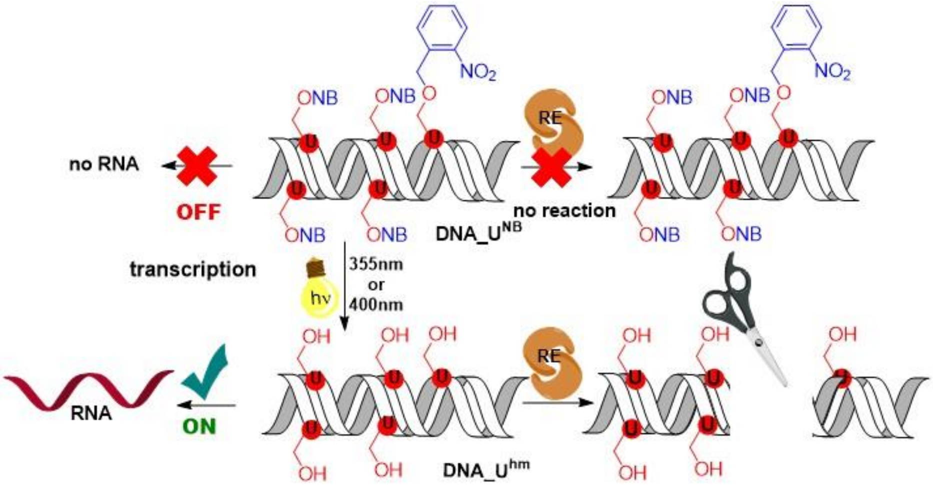

Scheme S2: *Study of transcription of HM- and NB- modified DNA*

All DNA samples used for a study of deprotection of nitrobenzyl- (NB-) modification on modified DNA were synthesized according the reported protocols (chapter *2.2.1* - *2.2.3*) and purified with Agencourt AMPure XP magnetic beads.

Firstly, the cleavage of nitrobenzyl modification from NB-modified DNA was studied on DNA synthesized in the presence of **dU^{NB}TP**. The NB-modified DNA was irradiated by UV lamps of different intensities, with the maximum absorbance at 355 nm or 400 nm. The degree of deprotection of NB-modified DNA was controlled with cleavage by restriction endonuclease - RsaI. Then, the irradiated DNA was used as a template for an *in vitro* transcription assay. The experiments were performmed with *NON*-labelled DNA samples, and after optimization of condition for deprotection, the experiments were repeated with *^{32}P*-labelled DNA under the conditions in which we observed the best results.

4.2.1. Kinetic study of deprotection of NB-modified DNA irradiated by UV lamps at either 355 nm (1 mW) or 400 nm (25 mW)

The purified NB-modified DNA was diluted to the final concentration of approx. 20 ng/ µL (according NanoDrop) and used as a stock for irradiation experiments. Approx. 240 ng of stock NB-modified DNA was irradiated by UV lamps (either 355nm or 400nm) at following time

intervals 15 min / 60 min / 120 min / 300 min. The samples were irradiated either in the presence of 2 µL H_2O (to reach the same dilution as in the case of samples irradiated in the presence of additives) or in the presence of additives (1 µL of (50 mM) DTT and 1 µL of (1 mM) NaN_3). The irradiation of samples was performed in cold room (+7-9 °C) because the long time interval of irradiation. The degree of deprotection of NB-modified DNA was controlled with cleavage by restriction endonuclease – RsaI (1.4 ul for 100 ng of DNA). (***Figure S4***) Then, the irradiated DNA was used as a template for an *in vitro* transcription assay (***Figure S5***).

A)

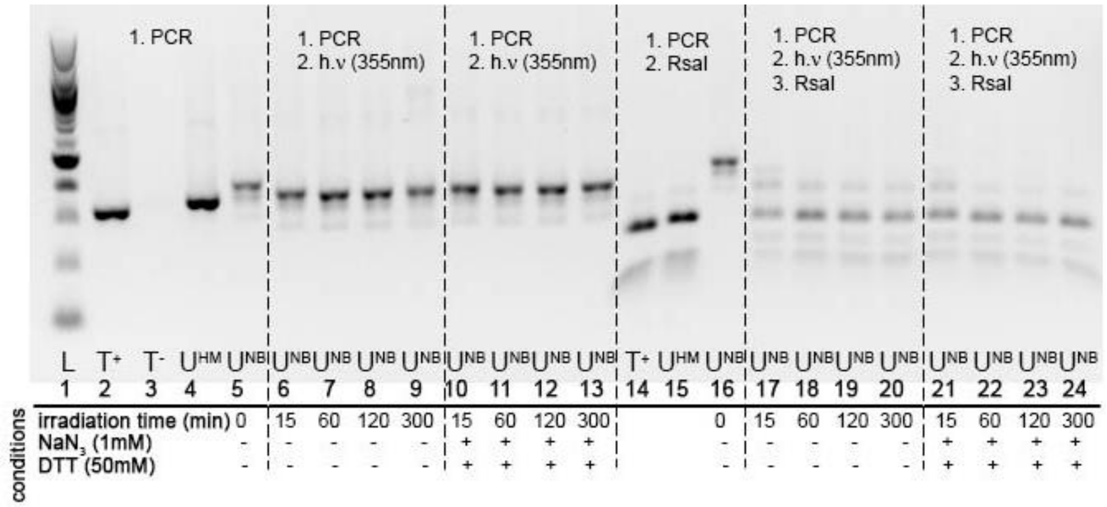

B):

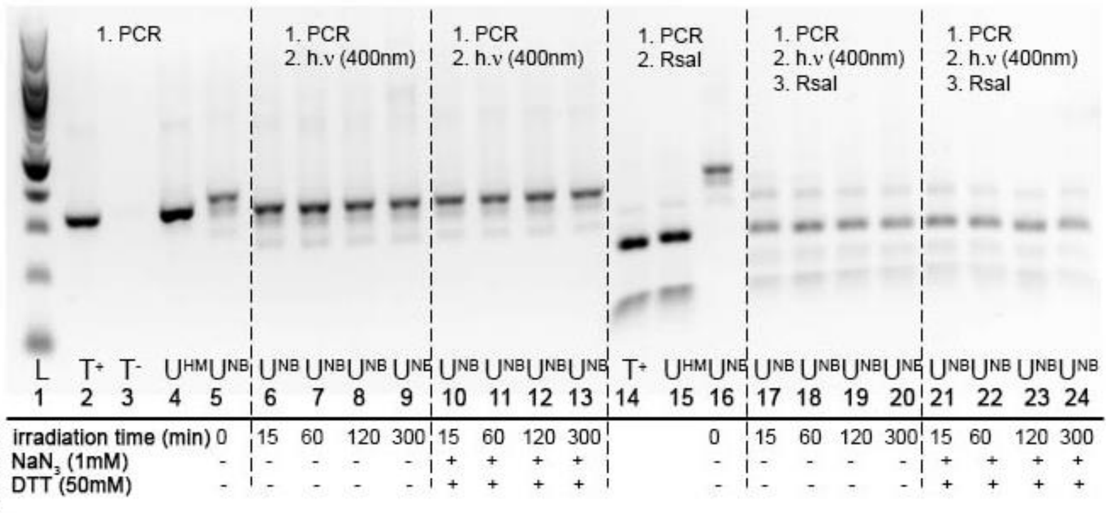

Figure S4: Kinetic study of deprotection of NB-modified DNA irradiated by UV lamps **A**) 355 nm or **B**) 400 nm: In gels, the order of samples is as follows: lanes 1 (**L**): ladder (mix of commercial dsDNA with specific length); lanes 2 (**T⁺**): PCR was run in the presence of natural dNTPs; lanes 3 (**T⁻**): PCR was run in the presence of dCTP, dGTP, dATP; lanes 4, (**U^{HM}**): PCR was run in the presence of three natural dNTPs (dCTP, dGTP, dATP) and **$dU^{hm}TP$**; lanes 5, (**U^{NB}**): PCR was run in the presence of three

natural dNTPs (dCTP, dGTP, dATP) and **dU^{NB}TP**; lanes 14 (**T**$^{+}$): natural PCR product cleaved by RsaI; lanes 15 (**U**^{HM}): hm-modified DNA cleaved by RsaI Lanes 16, (**U**^{NB}): NB-modified DNA cleaved by RsaI**.** In gel **A):** lanes 6-9: **U**^{NB} modified DNA without additives irradiated by UV lamp -355nm at the following time intervals: 15/ 60/ 120/ 300 min; lanes 17-20: **U**^{NB} modified DNA without additives irradiated by UV lamp -355nm at the following time intervals: 15/ 60/ 120/ 300 min and cleaved by RsaI. lanes 10-13: **U**^{NB} modified DNA in the presence of additives irradiated by UV lamp – 355 nm at the following time intervals: 15/ 60/ 120/ 300 min; lanes 21-24: **U**^{NB} modified DNA in the presence of additives irradiated by UV lamp – 355 nm at the following time intervals: 15/ 60/ 120/ 300 min and cleaved by RsaI. In gel **B):** lanes 6-9: **U**^{NB} modified DNA without additives irradiated by UV lamp -400 nm at the following time intervals: 15/ 60/ 120/ 300 min; lanes 17-20: **U**^{NB} modified DNA without additives irradiated by UV lamp -400 nm at the following time intervals: 15/ 60/ 120/ 300 min and cleaved by RsaI. lanes 10-13: **U**^{NB} modified DNA in the presence of additives irradiated by UV lamp – 400 nm at the following time intervals: 15/ 60/ 120/ 300 min; lanes 21-24: **U**^{NB} modified DNA in the presence of additives irradiated by UV lamp – 400 nm in time intervals 15/ 60/ 120/ 300 min and cleaved by RsaI.

4.2.1.1. Transcription data for kinetic study of deprotection of NB-modified DNA irradiated by UV lamps either 355 nm (1 mW) or 400 nm (25 mW)

Multiple-round *in vitro* transcriptions were carried out under the general conditions (*see Section 4.1.*). Also the quantification of relative transcriptions was performed in the same way as described above.

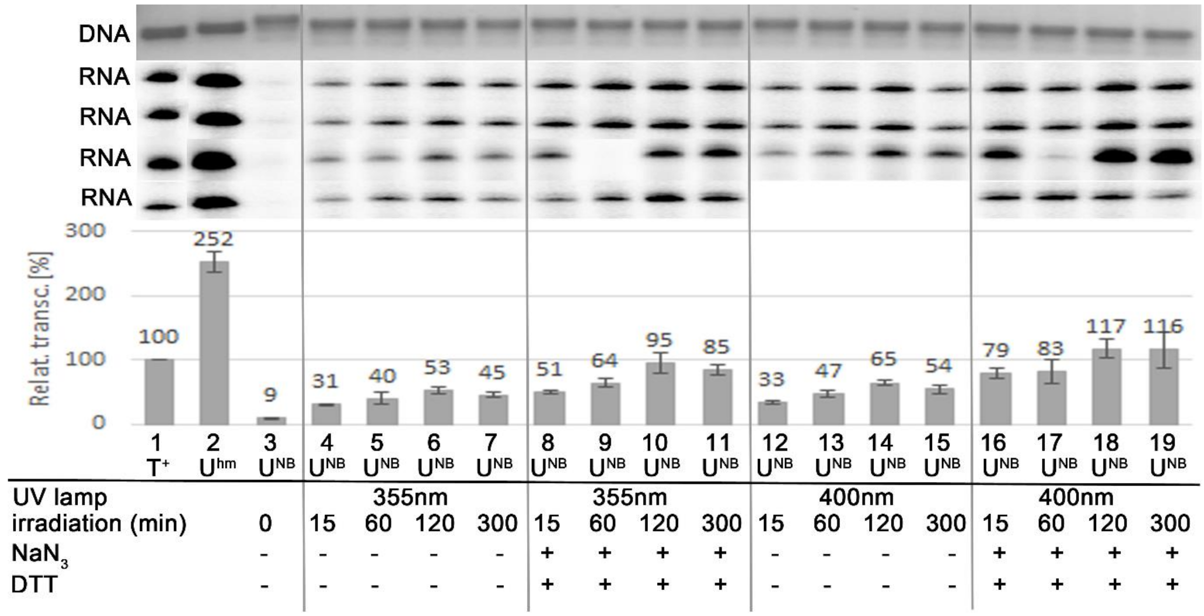

Figure S5: DNA templates prepared under *Procedure 4.2.1.*and their transcription

4.2.1.2. The effect of additives (NaN_3 and DTT) on deprotection of NB-modified DNA

The purified NB-modified DNA was diluted to the final concentration of approx. 20 ng/ µL (according NanoDrop) and used as a stock for irradiation experiments. Approx. 240 ng of stock NB-modified DNA was irradiated by UV lamp (400 nm) at the following time intervals: 15 min or 60 min. The samples were irradiated either in the presence of 2 µL H_2O (to reach the same dilution as in the case of samples irradiated in the presence of additives) or in the presence of additives (1 µL of (50 mM) DTT and 1 µL of (1 mM) NaN_3). The irradiation of samples was performed at room temperature. The irradiated DNA was used as a template for an *in vitro* transcription assay. (***Figure S6***) Multiple-round *in vitro* transcriptions were carried out under the general conditions (*see Section 4.1.*). Two independent experiments were performed. The quantification of relative transcriptions was also performed in the same way as above.

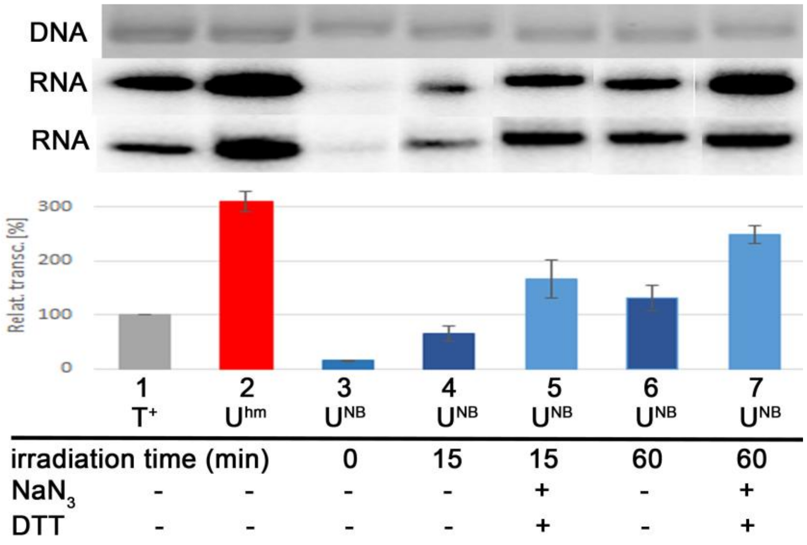

Figure S6: DNA templates prepared under *Procedure 4.2.1.2.*and their transcription

4.2.2. Kinetic study of deprotection of NB-modified DNA irradiated by UV lamps 365 nm (0.8 W)

The purified NB-modified DNA was diluted to the final concentration of approx. 20 ng/µL (according NanoDrop) and used as a stock for irradiation experiments. Approx. 240 ng of stock NB-modified DNA was irradiated by UV lamp (365 nm) at the following time intervals: 5 min / 10 min / 20 min / 30 min. The samples were irradiated either in the presence of 2 µL H_2O (to reach the same dilution as in the case of samples irradiated in the presence of additives) or in the presence of additives (1 µL of (1 mM) NaN_3 and 1 µL of (50 mM) DTT or 1 µL of (100 mM)

DTT). The irradiation of samples was performed at room temperature. The degree of deprotection of NB-modified DNA was controlled with cleavage by restriction endonuclease – RsaI (1.4 ul for 100 ng of DNA). (***Figure S7***) Then, the irradiated DNA was used as a template for an *in vitro* transcription assay (***Figure S8***).

A)

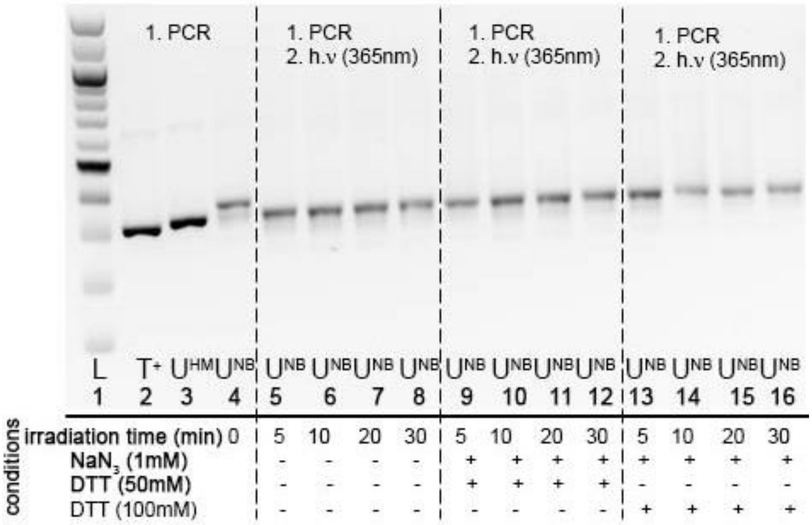

B)

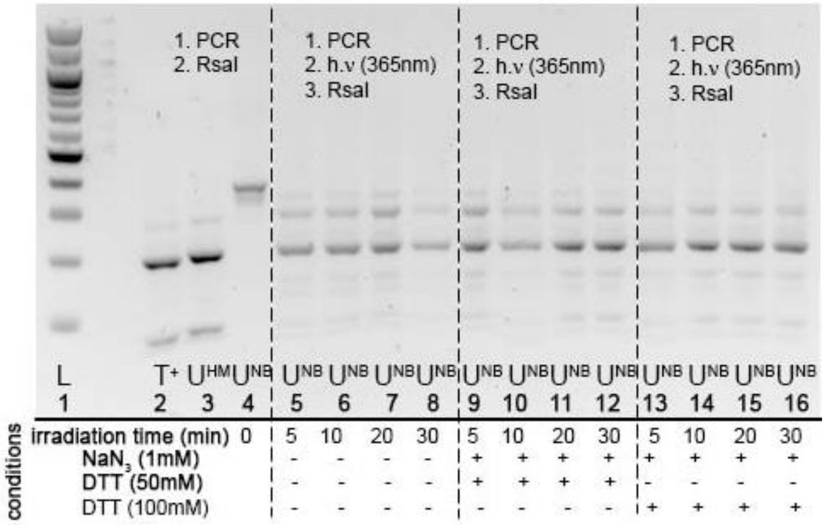

Figure S7: Kinetic study of deprotection of NB-modified DNA irradiated by UV lamp 365 nm***:*** In gel **A)**: all samples on a gel are right after PCR reaction or irradiation by UV. In gel **B)**: all samples on a gel are PCR products or samples after irradiation cleaved by restriction enzyme-RsaI. In gels, the order of samples is as follows: lanes 1 (**L**): ladder (mix of commercial dsDNA with specific length); lanes 2 (**T^{+}**): PCR was run in the presence of natural dNTPs; lanes 3 (**U^{HM}**): PCR was run in the presence of three natural dNTPs (dCTP, dGTP, dATP) and **dU^{hm}TP**; lanes 4 (**U^{NB}**): PCR was run in the presence of three natural dNTPs (dCTP, dGTP, dATP) and **dU^{NB}TP**; lanes 5-8: **U^{NB}** modified DNA without additives

irradiated by UV lamp -365 nm at the following time intervals: 5/ 10/ 20/ 30 min; lanes 9-12: **U^{NB}** modified DNA in the presence of additives (50 mM DTT and 1 mM NaN_3) irradiated by UV lamp -365nm at the following time intervals: 5/ 10/ 20/ 30 min. lanes 13-16: **U^{NB}** modified DNA in the presence of additives (100 mM DTT and 1 mM NaN_3) irradiated by UV lamp – 365 nm at the following time intervals: 5/ 10/ 20/ 30 min.

4.2.2.1. Transcription data for kinetic study of deprotection of NB-modified DNA irradiated by UV lamps 365 nm (0.8 W)

Multiple-round *in vitro* transcriptions were carried out under the general conditions (*see Section 4.1.*). Also the quantification of relative transcriptions was performed in the same way as described above.

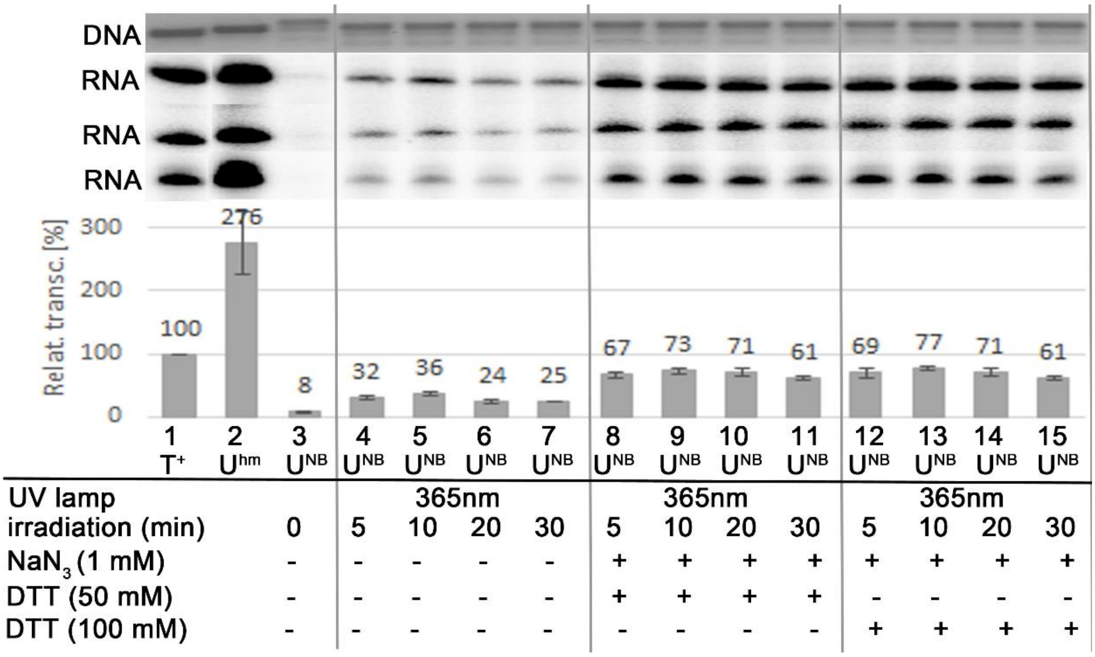

Figure S8: DNA templates prepared under *Procedure 4.2.2.*and their transcription

4.2.3. Kinetic study of deprotection of U^{NB}-modified DNA irradiated by UV lamp 400 nm (3 W)

The purified *32P*- labelled NB-modified DNA was diluted to the final concentration of approx. 20 ng/ µL (according NanoDrop) and used as a stock for irradiation experiments. Approx. 240 ng of stock NB-modified DNA was irradiated by UV lamp (400 nm) at the following time intervals: 5 min / 10 min / 20 min / 30 min / 35 min / 40 min / 45 min / 50 min / 60 min. The samples were irradiated in the presence of additives (1 µL of (1 mM) NaN_3 and 1 µL of (50

mM) DTT) in 1.5 ml Eppendorf tubes at room temperature. The sides of the Eppendorf tubes were covered with alu-foil and samples were irradiatiated from the top of the opened tube. The degree of deprotection of NB-modified DNA was controlled with cleavage by restriction endonuclease – RsaI (1.4 ul for 100 ng of DNA). (***Figure S9***) Then, the irradiated DNA was used as a template for an *in vitro* transcription assay. (***Figure S10***, ***Figure 1***)

A)

B)

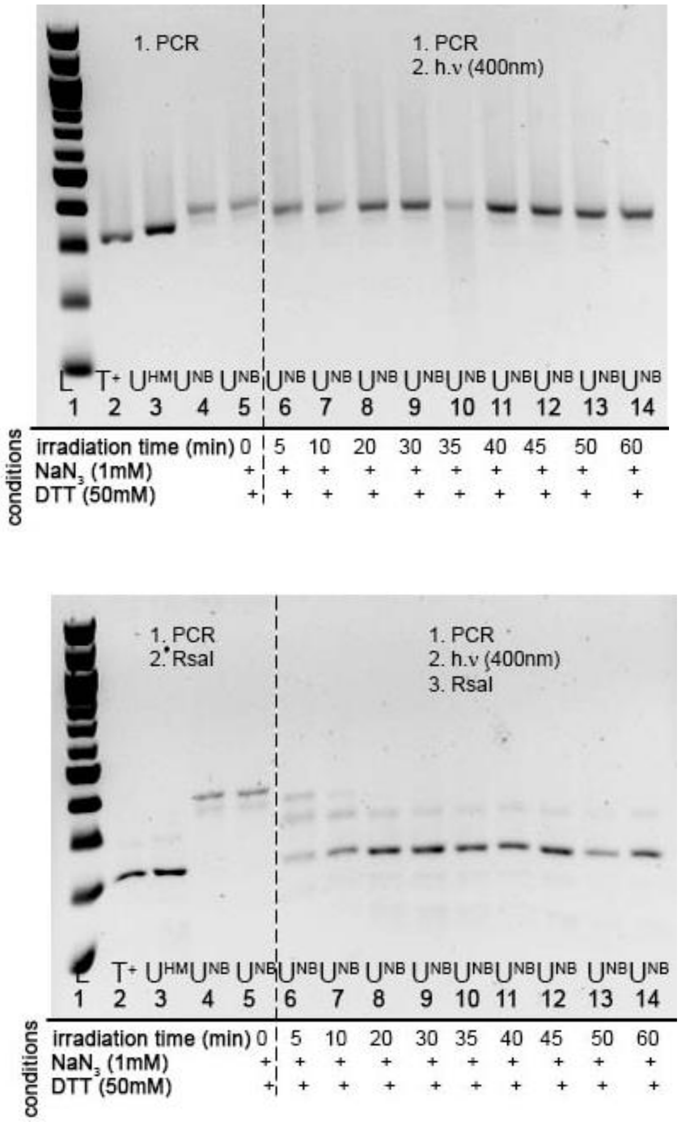

Figure S9: Kinetic study of deprotection of U^{NB}-modified DNA irradiated by UV lamp 400 nm***:*** In gel **A)**: all samples on a gel are right after PCR reaction or irradiation by UV. In gel **B)**: all samples on a gel are PCR products or samples after irradiation cleaved by restriction enzyme-RsaI. In gels, the order of samples is as follows: lanes 1 (**L**): ladder (mix of commercial dsDNA with specific length); lanes 2 (**T^{+}**): PCR was run in the presence of natural dNTPs; lanes 3 (**U^{HM}**): PCR was run in the presence of three natural dNTPs (dCTP, dGTP, dATP) and **dU^{hm}TP**; lanes 4

(U^{NB}): PCR was run in the presence of three natural dNTPs (dCTP, dGTP, dATP) and **dU^{NB}TP**; lanes 5 (U^{NB}): U^{NB} modified DNA in the presence of additives; lanes 6-14: U^{NB} modified DNA in the presence of additives (50 mM DTT and 1 mM NaN_3) irradiated by UV lamp -400 nm at following time intervals: 5/ 10/ 20/ 30 / 35 /40 /45 /50 /60 min.

4.2.3.1. Transcription data for kinetic study of deprotection of DNA_U^{NB} irradiated by UV lamp 400 nm (3 W)

Multiple-round *in vitro* transcriptions were carried out under the general conditions (*see Section 4.1.*). For the quantification of relative transcriptions the transcript signals (background was subtracted) were normalized to the average of DNA template signals. Subsequently, signals of transcriptions of non-modified and modified DNA templates were normalized to the signal of natural DNA (T^+), which was set as 100 % (T^+ - transcription of nonmodified DNA diluted in water). At least three independent experiments were performed.

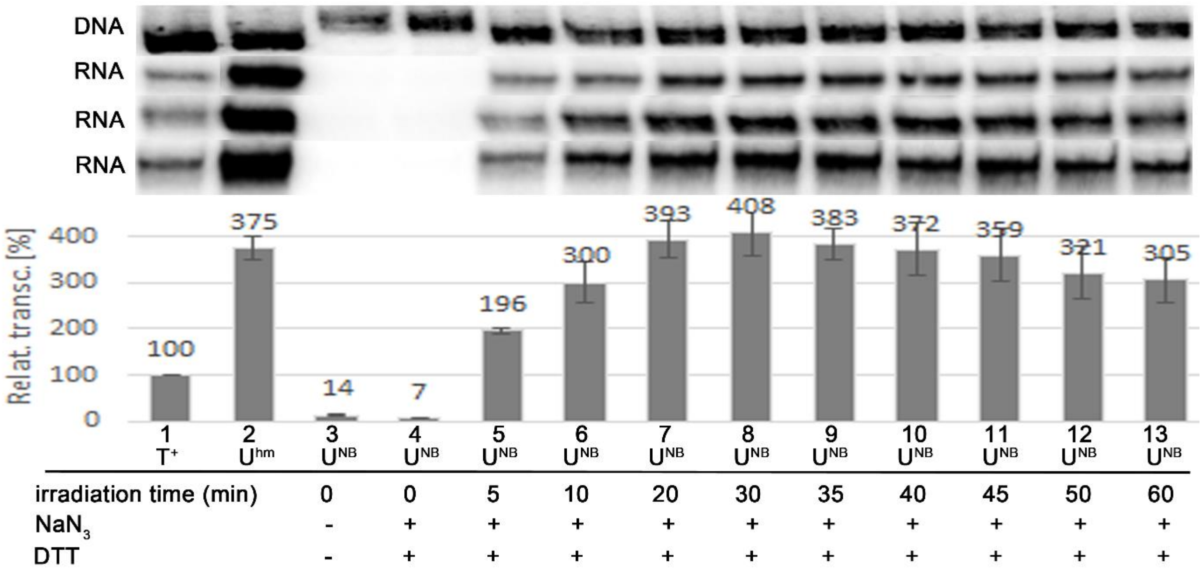

Figure S10: ^{32}P-labelled DNA templates prepared under *Procedure 4.2.3.* and their transcription

4.2.4. Kinetic study of deprotection of C^{NB}-modified DNA irradiated by UV lamp 400nm (3W)

The purified ^{32}P-labelled NB-modified DNA synthesized in the presence of dC^{NB}TP was diluted to the final concentration of approx. 20 ng/ µL (according NanoDrop) and used as a stock for irradiation experiments. Approx. 240 ng of stock NB-modified DNA was irradiated by UV lamp

(400 nm) in time intervals 2 min / 5 min / 7 min/ 10 min / 15 min / 17 min / 20 min / 30 min / 40 min . The samples were irradiated in the presence of additives (1 µL of (1 mM) NaN_3 and 1 µL of (50 mM) DTT in 1.5 mL Eppendorf tubes at room temperature. The sides of the Eppendorf tubes were covered with alu-foil and samples were irradiatiated from the top of the opened tube. The degree of deprotection of NB-modified DNA was not controlled with cleavage by restriction endonuclease, because even small hydroxymethyl modification is not tolerated with restriction enzymes –RsaI and AluI (*see chapter* 3.) (***Figure S11***) Then, the irradiated DNA was used as a template for an *in vitro* transcription assay (***Figure S12***).

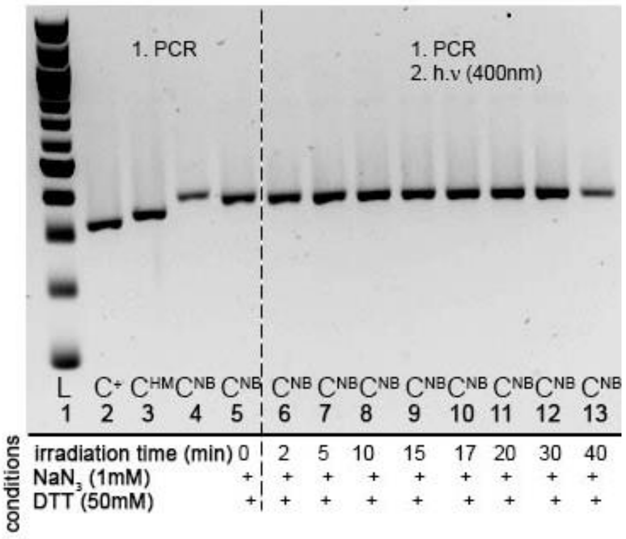

Figure S11: Kinetic study of deprotection of C^{NB}-modified DNA irradiated by UV lamp 400nm: In a gel, all samples on the gel are right after PCR reaction or irradiation by UV. The order of samples is as follows: Lanes 1 (**L**): ladder (mix of commercial dsDNA with specific length); lanes 2 (**C⁺**): PCR was run in the presence of natural dNTPs; lanes 3 ($\mathbf{C^{HM}}$): PCR was run in the presence of three natural dNTPs (dTTP, dGTP, dATP) and $\mathbf{dC^{hm}TP}$; lanes 4 ($\mathbf{C^{NB}}$): PCR was run in the presence of three natural dNTPs (dTTP, dGTP, dATP) and $\mathbf{dC^{NB}TP}$; lanes 5 ($\mathbf{C^{NB}}$): $\mathbf{C^{NB}}$ modified DNA in the presence of additives; lanes 6-14: $\mathbf{C^{NB}}$ modified DNA in the presence of additives (50 mM DTT and 1 mM NaN_3) irradiated by UV lamp -400 nm at the following time intervals: 2 /5/ 10/15 /17 /20 /30 /40 min.

4.2.4.1. Transcription data for kinetic study of deprotection of DNA_C^{NB} irradiated by UV lamp 400 nm (3 W)

Multiple-round *in vitro* transcriptions were carried out under the general conditions (*see Section 4.1.*). For the quantification of relative transcriptions the transcript signals (background was subtracted) were normalized to the average of DNA template signals. Subsequently, signals of transcriptions of non-modified and modified DNA templates were normalized to the signal of natural DNA (C^+), which was set as 100 % (C^+ - transcription of nonmodified DNA diluted in water). Two independent experiments were performed.

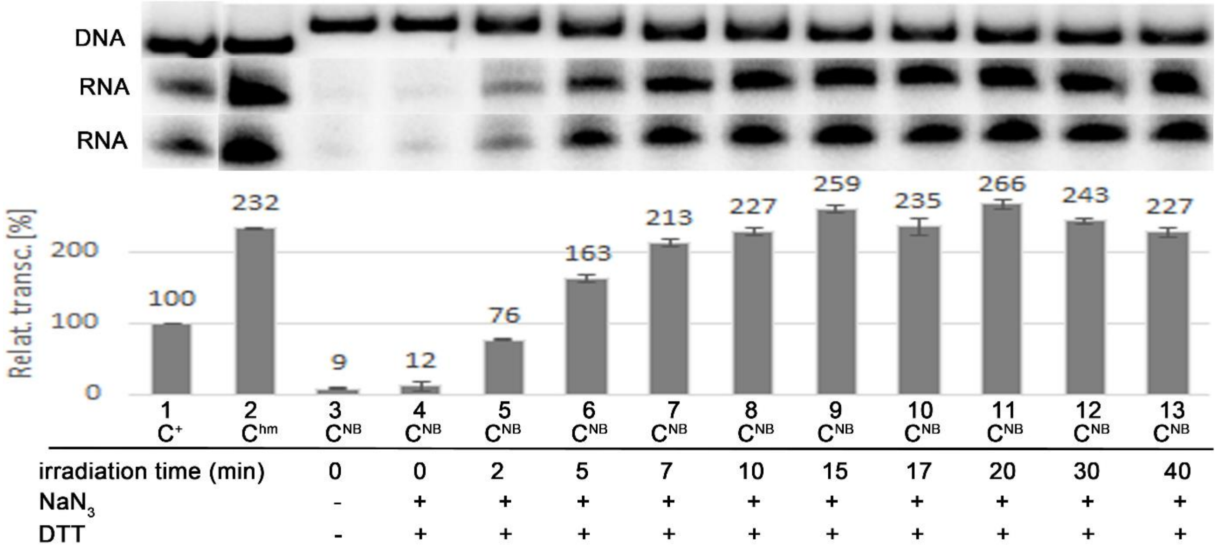

Figure S12: *³²P*-labelled DNA templates prepared under *Procedure 4.2.4.* and their transcription

5. Study of enzymatic phosphorylation of U^{hm} modified DNA

5.1. Model study of phosphorylation by cleavage with REs

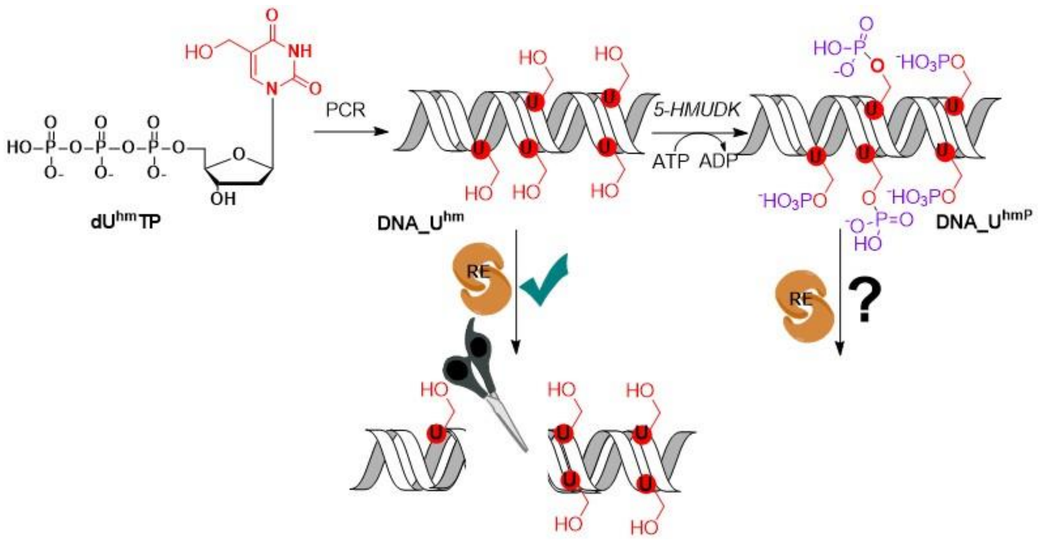

Scheme S3: Study of phosphorylation by cleavage with REs

HM-modified DNA synthesized in the presence of **dU^{hm}TP** and after purification was incubated with 5-HMUDK (5-hydroxymethyluridine DNA Kinase) at 37°C for 30 min. For the first study, reactions with different volumes of 5-HMUDK (20U; 1.2 µL or 18U; 0.9 µL or 12U; 0.6 µL) were set up with HM-modified DNA. Natural DNA was also incubated under the same conditions as a control reaction. The samples after reaction were purified with Agencourt AMPure XP magnetic particles. We tried to verify the conversion of phosphorylation of hm-modified DNA by cleavage with restriction endonucleases – AluI or RsaI. However AluI tolerated and cleaved also the phosphorylated DNA. On the other hand, RsaI has two restriction sites in the DNA template and we observed only partial cleavage of the phosphorylated DNA at one of them (***Figure S13***). Therefore, the RE cleavage could not be reliably used for determination of the outcome of phosphorylation and we directly proceeded to the transcription study.

A)

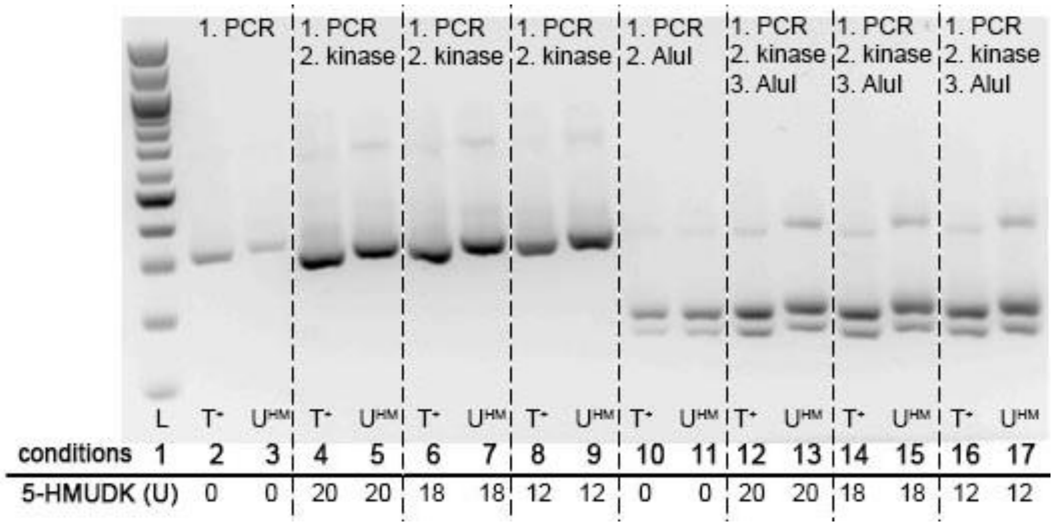

B)

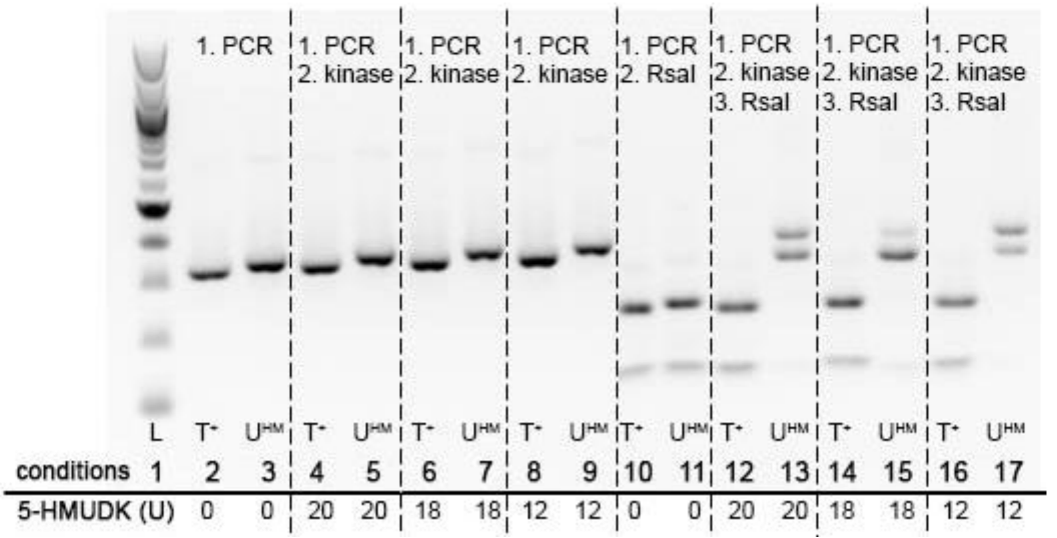

Figure S13: Study of cleavage phosphorylated DNA by restriction enzyme AluI or RsaI***:*** In gels, all samples on the gel are right after PCR reaction or after incubation with 5-HMUDK. In the gel **A)** the restriction enzyme used for study of cleavage of DNA was AluI. In the gel **B)** the restriction enzyme used for study of cleavage of DNA was RsaI. The order of samples is as follows: Lanes 1 (**L**): ladder (mix of commercial dsDNA with specific length); lanes 2 (T^+): PCR was run in the presence of natural dNTPs; lanes 3 (**U^{HM}**): PCR was run in the presence of three natural dNTPs (dCTP, dGTP, dATP) and **dU^{hm}TP**; lanes 4: natural DNA after incubation with 20U of 5-HMUDK; lanes 5: hm-modified DNA after incubation with 20 U of 5-HMUDK; lanes 6: natural DNA after incubation with 18U of 5-HMUDK; lanes 7: hm-modified DNA after incubation with 18U of 5-HMUDK; lanes 8: natural DNA after incubation with 12U of 5-HMUDK; lanes 9: hm-modified DNA after incubation with 12U of 5-HMUDK; lanes 10: natural DNA cleaved by restriction enzyme; lanes 11: hm-modified DNA cleaved by restriction enzyme; lanes 12: natural DNA after incubation with 20U of 5-HMUDK cleaved by restriction enzyme; lanes 13: hm-modified DNA after incubation with 20U of 5-HMUDK cleaved by restriction enzyme; lanes 14: natural

DNA after incubation with 18U of 5-HMUDK cleaved by restriction enzyme; lanes 15: hm-modified DNA after incubation with 18U of 5-HMUDK cleaved by restriction enzyme; lanes 16: natural DNA after incubation with 12U of 5-HMUDK cleaved by restriction enzyme; lanes 17: hm-modified DNA after incubation with 12U of 5-HMUDK cleaved by restriction enzyme.

5.2. Transcription study for DNA_U^{hm} phosphorylated with 5-HMUDK

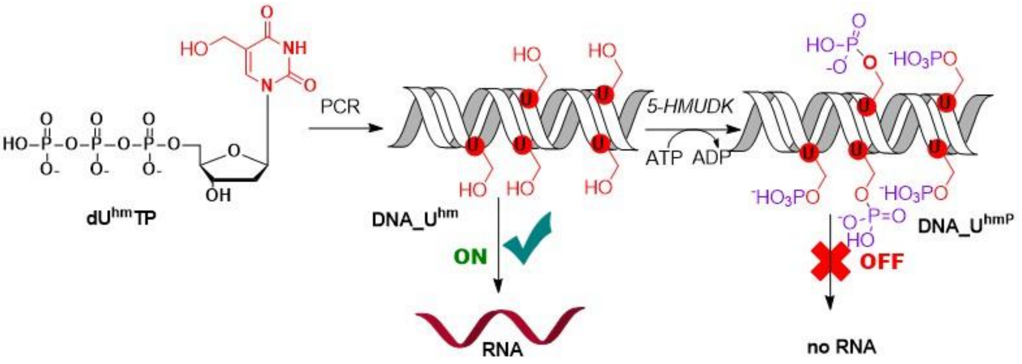

Scheme S4: Study of transcription for phosphorylated DNA_U^{hm}

Multiple-round *in vitro* transcriptions were carried out under the general conditions (*see Section 4.1.*). The quantification of relative transcriptions was performed in the same way as above. The results are shown in ***Figure S14***.

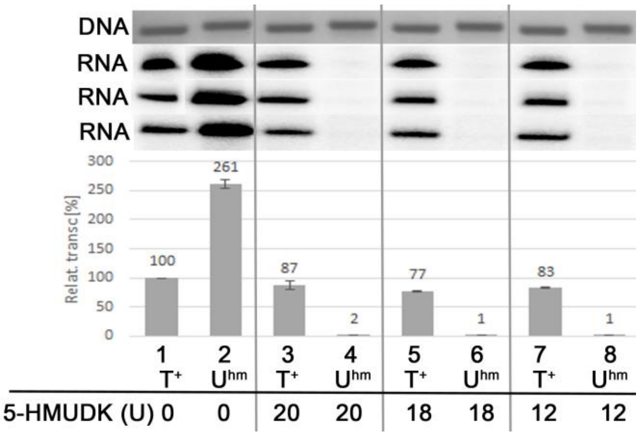

Figure S14: DNA templates prepared under procedure described in *section 5.* and their transcription

6. Selective phosphorylation of NB-modified DNA irradiated by UV

A)

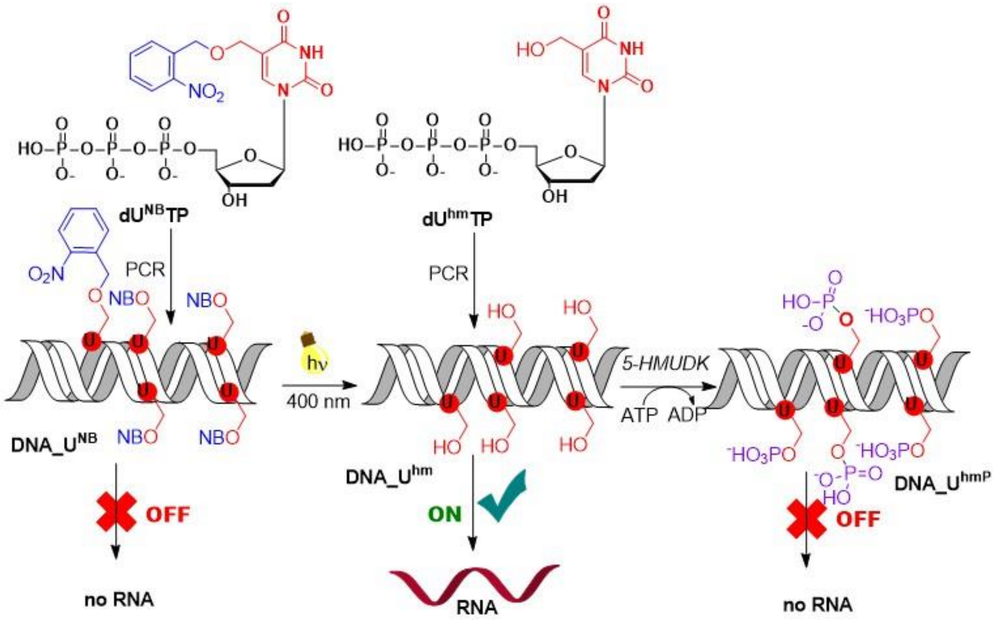

B)

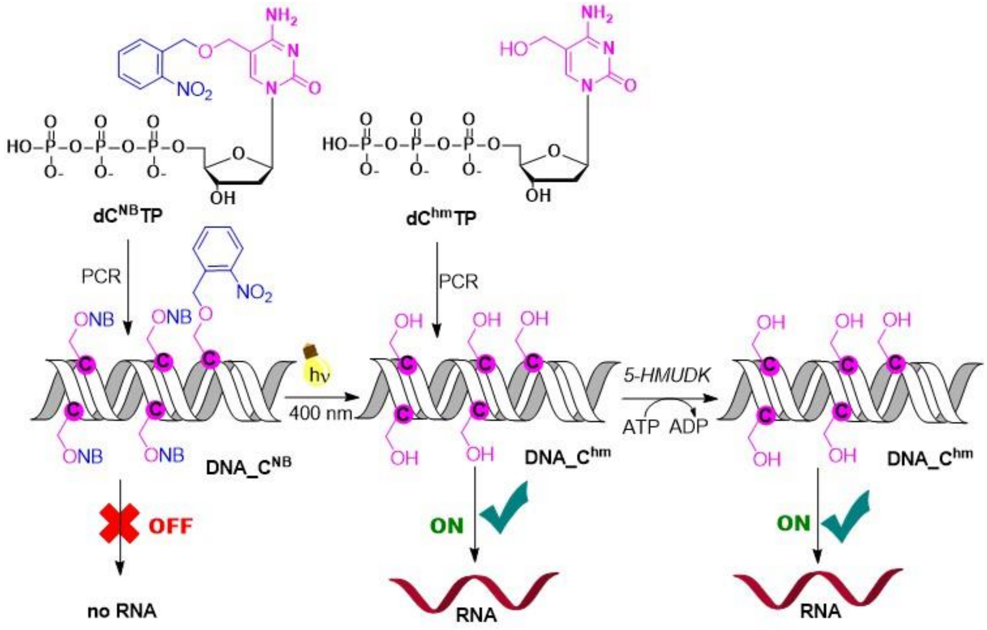

Scheme S5: Switching ON and OFF of transcription **A)** for U^X-modified DNA **B)** for C^X-modified DNA (X=hm or NB)

^{32}P-labelled DNA was synthesized by PCR and purified with Ampure magnetic beads. U^{NB}-modified **DNA_U^{NB}** (cca 240 ng) was irradiated in the presence of additives (1 µL of (1 mM) NaN_3 and 1 µL of (50 mM) DTT) with UV lamp (3 W, 400 nm) during 30 min at room temperature (***Scheme S5A***, ***Figure S15A*** sample no. 10). The C^{NB-}modified DNA was under the

same conditions irradiated during 10 min. The irradiation experiments were in each case repeated in six portions. After irradiation, all six portions were mixed together and non purified previously irradiated DNA (400 ng) was incubated with 5-HMUDK (0.3 µL) in 1X T4 DNA Ligase Reaction Buffer at 37°C for 30min (***Scheme S5A, B; Figure S15A, B*** sample no. 12).

As a control for phoshorylation, non-irradiated natural DNA and hydroxymethyl-modified DNA (synthesized by PCR in the presence of either **dU^{hm}TP** or **dC^{hm}TP**) were also incubated with 5-HMUDK under the same conditions (***Scheme S5A, B; Figure S15A, B*** samples no. 4 and 8) .

As a control reactions for irradiation, natural and hydroxymethyl- modified DNA (cca 240ng) were irradiated in the presence of additives (1 µL of (1 mM) NaN_3 and 1 µL of (50 mM) DTT) with UV lamp (3 W, 400 nm) during appropriate time intervals (30 min for U-set and 10 min for C-set) at room temperature (***Scheme S5A, B; Figure S15A, B*** samples no.2 and no. 6).

The irradiated NB-modified samples, natural and hydroxymethyl- modified DNA samples right after PCR (without irradiation prepared according procedure *2.3.1. - 2.3.3.*) were mixed with 1X T4 DNA Ligase Reaction Buffer to control for its influence on transcription (***Scheme S5A, B; Figure S15A, B*** samples no. 3, no. 7, no. 11).

All samples were diluted to the final concentration of 5 ng/ µL used as a templates for study of transcription experiments.

A)

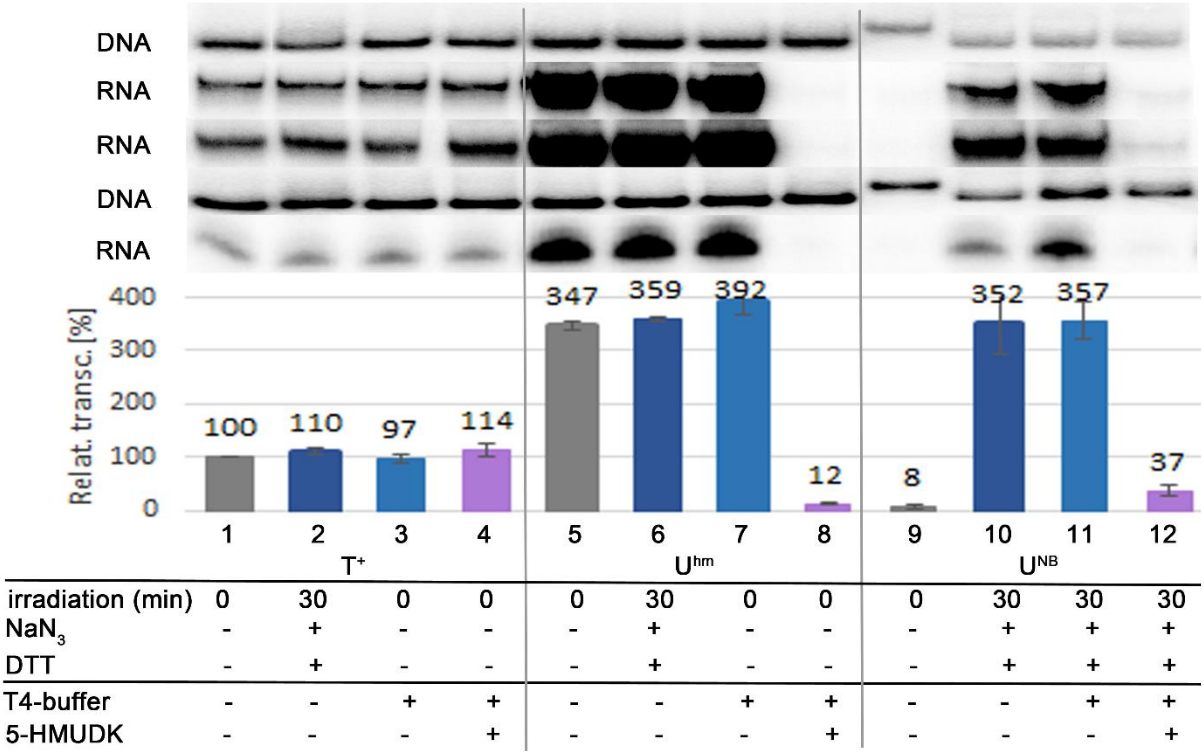

B)

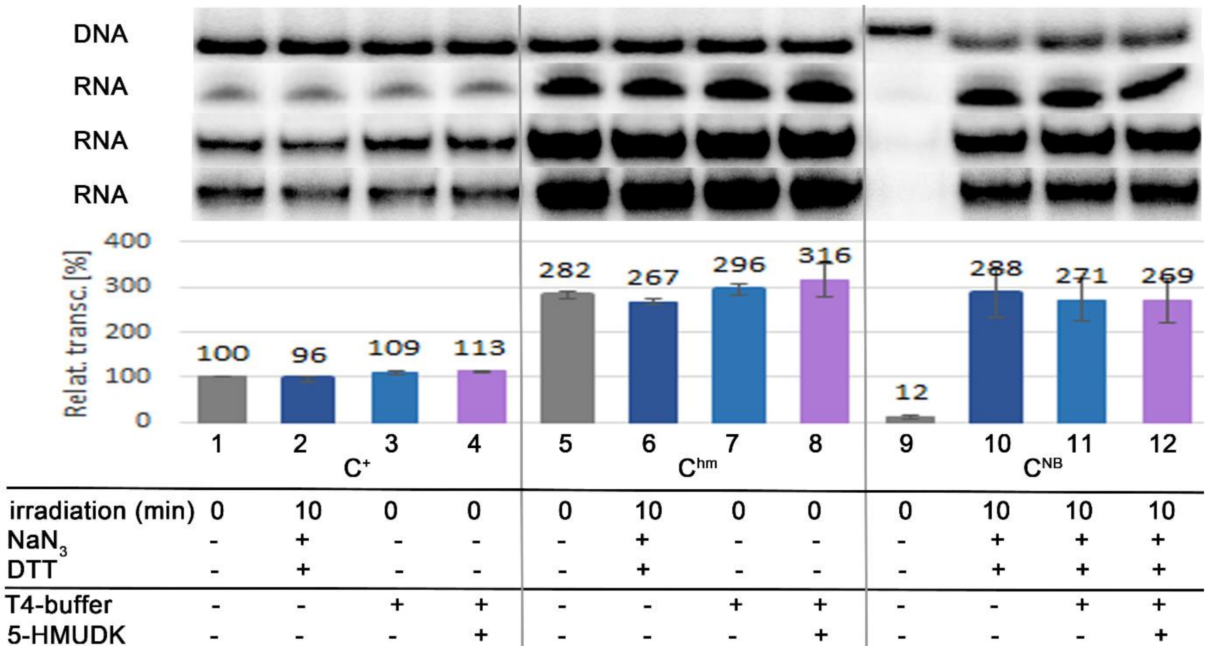

irradiation (min)	0	10	0	0	0	10	0	0	0	10	10	10
NaN_3	-	+	-	-	-	+	-	-	-	+	+	+
DTT	-	+	-	-	-	+	-	-	-	+	+	+
T4-buffer	-	-	+	+	-	-	+	+	-	-	+	+
5-HMUDK	-	-	-	+	-	-	-	+	-	-	-	+

***Figure S15*: DNA templates prepared under *Procedure 6.* and their transcription **A)** for U^X-modified DNA **B)** for C^X-modified DNA (X=hm or NB)

7. Sequencing of PCR products:

The correct sequence of all PCR products was confirmed with LIGHTRUN tube Sequencing service (GATC Biotech AG, Germany) using standard Sanger sequencing. In the case of C^{NB} modified dsDNA, C^{NB} modified DNA was used as a template for their rePCR with natural dNTPs according to the protocol reported in the sections *2.3.* of the SI, and the resulting product was sent for sequencing.

The red underlined part shows exact match of sequenced DNA with expected sequence in the presence of reverse primer. Blue underlined part shows exact match of sequenced DNA with expected sequence in the presence of forward primer. Double, violet underlined part was correctly sequenced in the presence of either forward or reverse primer.

7.1. dU^{hm}-modified DNA

5’-TTCAGCCATATATCCTCTGGCTAATAGGACTACTTCTAATCTGTACGAGCAGATCCATACGCCTGG

ACAGGCAATCAGGCTAGAGGAATTC***TATTTGACAAAAATGGGCTCGTGTTGTACAATAAATGT***GTC

TAAGCTTGGGTCCCACCTGACCCCATGCCGAACTCAGAAGTGAAACGCCGTAGCGCCGATGGTAGTG

TGGGGTCTCCCCATGCGAGAGTAGGGAACTGCCAGGCATCAAATAAAACGAAAGGCTCAGTCGAAA

GACTGGGCCTTTCGTTTTATCTGTTGTTTGTCGGTGAACGCTCTCC-3´

Primary strand (exact match for 271 bp):

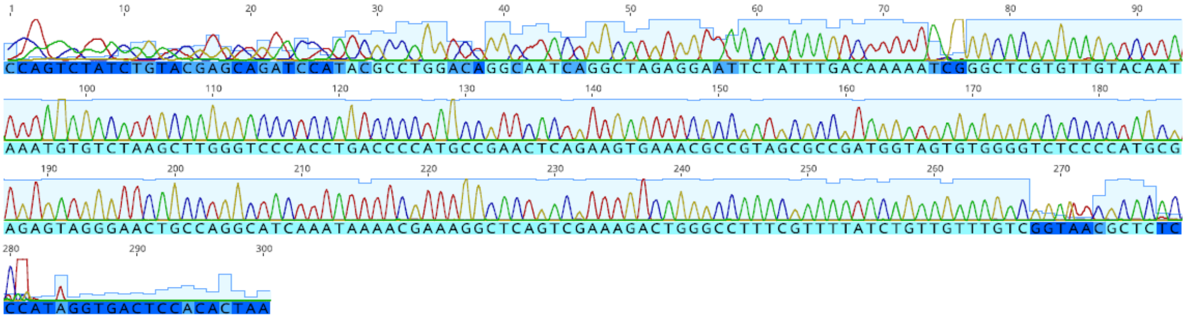

Complementary strand (exact match for 270 bp):

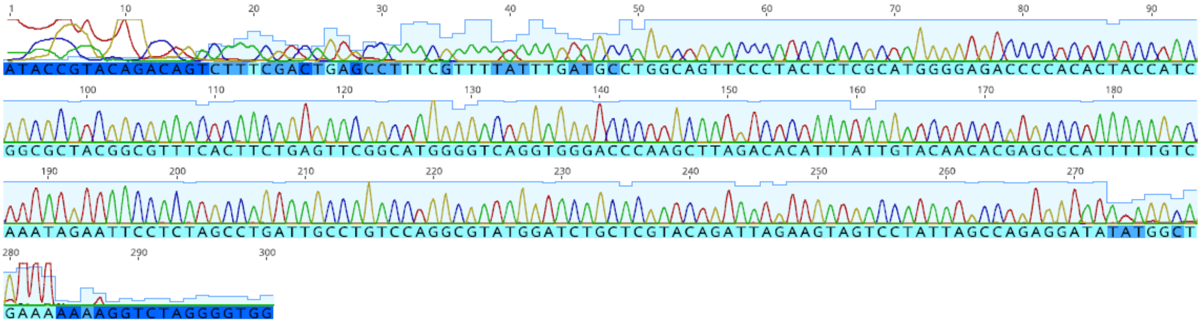

7.2. dU^{NB}-modified DNA

5’-TTCAGCCATATATCCTCTGGCTAATAGGACTACTTCTAATCTGTACGAGCAGATCCATACGCCTGG

ACAGGCAATCAGGCTAGAGGAATTC***TATTTGACAAAAATGGGCTCGTGTTGTACAATAAATGT***GTC

TAAGCTTGGGTCCCACCTGACCCCATGCCGAACTCAGAAGTGAAACGCCGTAGCGCCGATGGTAGTG

TGGGGTCTCCCCATGCGAGAGTAGGGAACTGCCAGGCATCAAATAAAACGAAAGGCTCAGTCGAAA

GACTGGGCCTTTCGTTTTATCTGTTGTTTGTCGGTGAACGCTCTCC-3´

Primary strand (exact match for 258 bp):

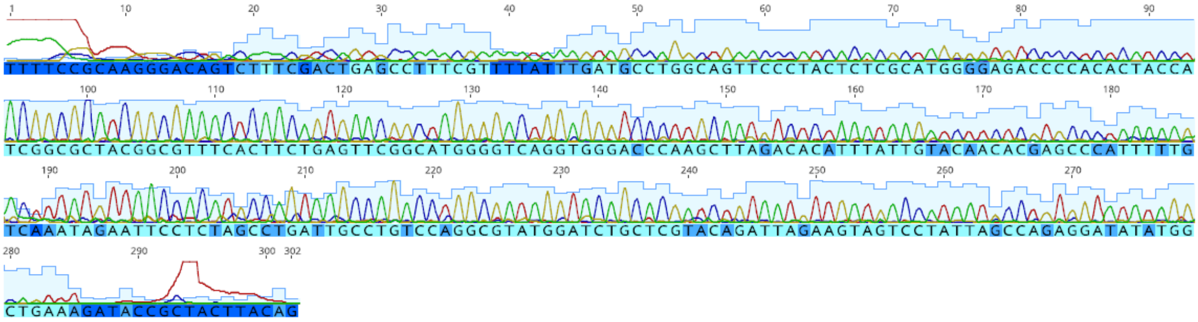

Complementary strand (exact match for 269 bp):

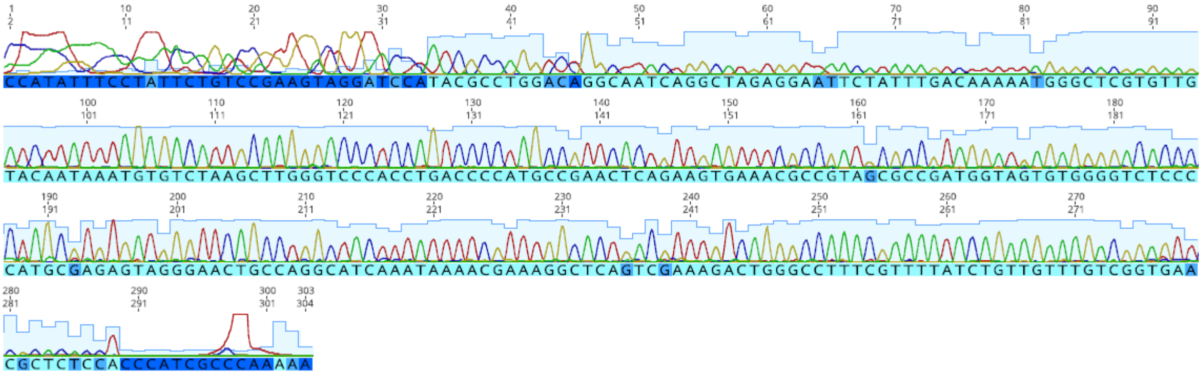

7.3. dC^{hm}-modified DNA

5’-TTCAGCCATATATCCTCTGGCTAATAGGACTACTTCTAATCTGTACGAGCAGATCCATACGCCTGG
ACAGGCAATCAGGCTAGAGGAATTC***TATTTGACAAAAATGGGCTCGTGTTGTACAATAAATGT***GTC
TAAGCTTGGGTCCCACCTGACCCCATGCCGAACTCAGAAGTGAAACGCCGTAGCGCCGATGGTAGTG
TGGGGTCTCCCCATGCGAGAGTAGGGAACTGCCAGGCATCAAATAAAACGAAAGGCTCAGTCGAAA
GACTGGGCCTTTCGTTTTATCTGTTGTTTGTCGGTGAACGCTCTCC-3´

Primary strand (exact match for 247 bp):

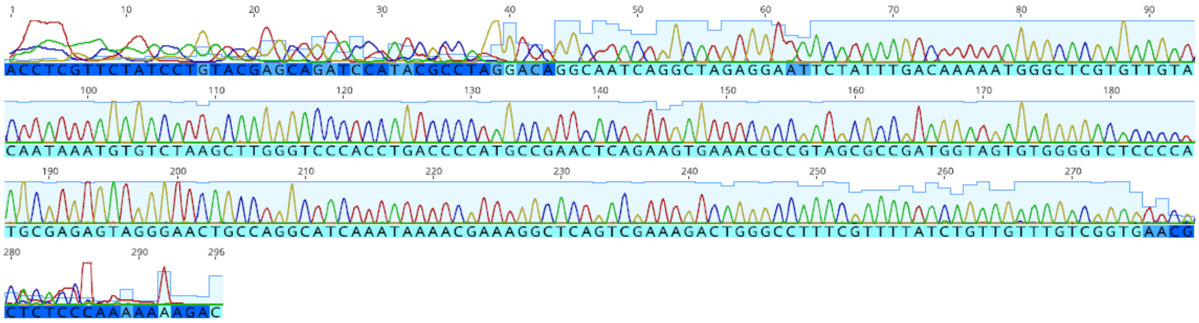

Complementary strand (exact match for 262 bp):

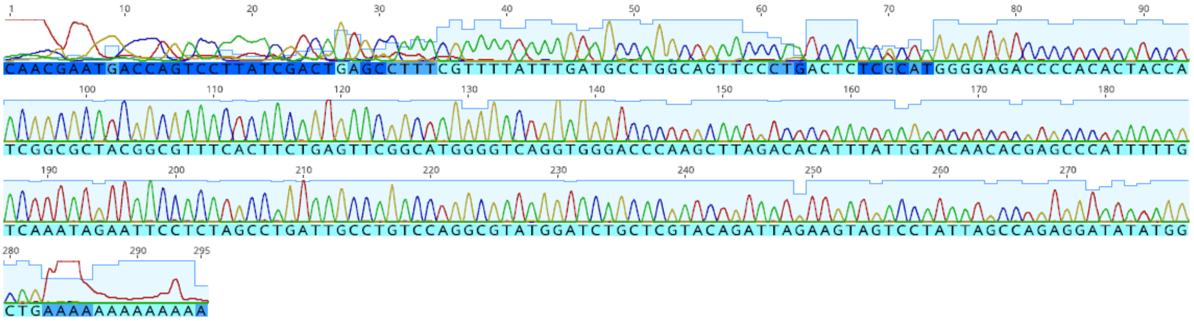

7.4. dC^{NB}-modified DNA

5’-TTCAGCCATATATCCTCTGGCTAATAGGACTACTTCTAATCTGTACGAGCAGATCCATACGCCTGG
ACAGGCAATCAGGCTAGAGGAATTC***TATTTGACAAAAATGGGCTCGTGTTGTACAATAAATGT***GTC
TAAGCTTGGGTCCCACCTGACCCCATGCCGAACTCAGAAGTGAAACGCCGTAGCGCCGATGGTAGTG
TGGGGTCTCCCCATGCGAGAGTAGGGAACTGCCAGGCATCAAATAAAACGAAAGGCTCAGTCGAAA
GACTGGGCCTTTCGTTTTATCTGTTGTTTGTCGGTGAACGCTCTCC-3´

Primary strand (exact match for 218 bp):

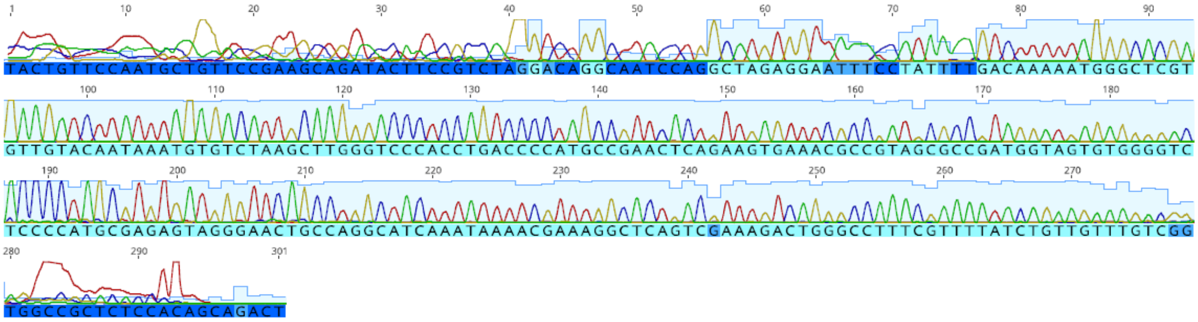

Complementary strand (exact match for 266 bp):

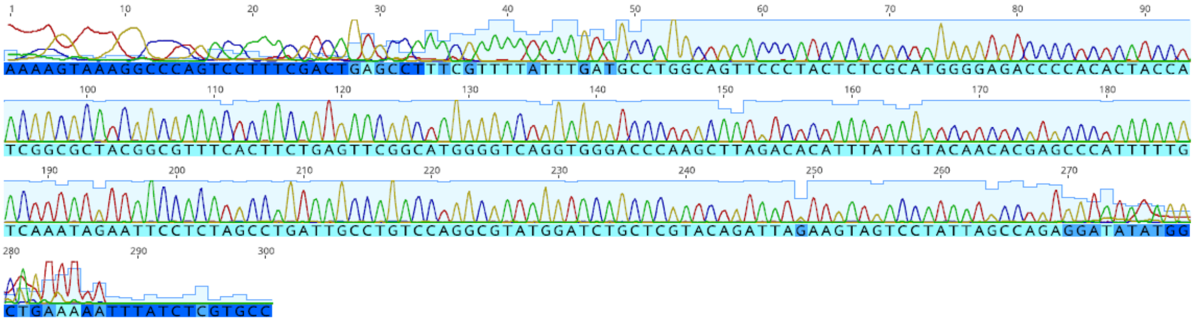

8. References

[1] Vaníková, Z.; Hocek, M. *Angew. Chem. Int. Ed.* **2014**, *53*, 6734–6737.

[2] Boháčová, S.; Vaníková, Z.; Poštová Slavětínská, L.; Hocek, M. *Org. Biomol. Chem.* **2018**, *16*, 5427–5432.

[3] Raindlová, V.; Janoušková, M.; Slavíčková, M.; Perlíková, P.; Boháčová, S.; Milisavljevič, N.; Šanderová, H.; Benda, M.; Barvík, I.; Krásný, L.; Hocek, M. *Nucleic Acids Res* **2016**, *44*, 3000–3012.

[4] Slavíčková, M.; Janoušková, M.; Šimonová, A.; Cahová, H.; Kambová, M.; Šanderová, H.; Krásný, L.; Hocek, M. Chem. Eur. J. 2018, 24, 8311–8314.

[5] Janoušková, M.; Vaníková, Z.; Nici, F.; Boháčová, S.; Vítovská, D.; Šanderová, H.; Hocek, M.; Krásný, L. *Chem. Commun.* **2017**, *53*, 13253-13255.
